# The blessing and curse of “no strings attached”: An automated literature analysis of psychological health and non-attachmental work in the digitalization era

**DOI:** 10.1371/journal.pone.0298040

**Published:** 2024-02-08

**Authors:** Lubna Rashid, Clemens Möckel, Stephan Bohn

**Affiliations:** 1 Chair of Entrepreneurship & Innovation Management (H76), Technische Universität Berlin, Berlin, Germany; 2 Independent Researcher, Berlin, Germany; 3 Humboldt Institute for Internet and Society, Berlin, Germany; 4 Department of Management, Freie Universität Berlin, Berlin, Germany; St John’s University, UNITED STATES

## Abstract

Amidst tremendous changes in the worlds of work in light of digitalization, non-attachmental work designs, where individuals gain income without being bound by a fixed administrative attachment to an employer, hold promises of self-actualization along with threats of insecurity. Today’s technology boom and the consequent flexibility and uncertainty it brings into workers’ lives may translate into inspiring growth opportunities or overloading pressure, contingent upon mental health and wellbeing impacts. This paper first provides a conceptualization of the non-attachmental work designs of the 21^st^ century, before proceeding to an extensive mapping of literature at their intersection with psychological health. This involves a machine-learning-driven review of 1094 scientific articles using topic modeling, combined with in-depth manual content analyses and inductive-deductive cycles of pattern discovery and category building. The resulting scholarly blueprint reveals several tendencies, including a prevalence of positive psychology concepts in research on work designs with high levels of autonomy and control, contrasted with narratives of disempowerment in service- and task-based work. We note that some psychological health issues are researched with respect to specific work designs but not others, for instance neurodiversity and the role of gender in ownership-based work, self-image and digital addiction in content-based work, and ratings-induced anxiety in platform-mediated task-based work. We also find a heavy representation of ‘heroic’ entrepreneurs, quantitative methods, and western contexts in addition to a surprising dearth of analyses on the roles of policy and technological interventions. The results are positioned to guide academics, decision-makers, technologists, and workers in the pursuit of healthier work designs for a more sustainable future.

## Introduction

“Our psychology is shaped by millions of years of genetic evolution, thousands of years of cultural evolution, and a short lifetime of experience” [1]. However, with the rise of globalization, connectivity, and digital technologies, work is continually and rapidly transitioning from stable to adaptable [2], making the role of this ‘short lifetime of experience’ in shaping worker psychological health and wellbeing evermore pronounced. Indeed, the frequency of major changes that a single modern-day human experiences has risen exponentially over the past few decades. In contrast with *nine-to-five* office employees, or even factory workers during the industrial revolution, farmers in the agrarian society, or hunter-gatherers before that, today’s workers wear several hats and may reinvent themselves and their work identities on a regular basis. The digital revolution led to a decline in steady, lifelong careers and trades [3,4], while catalyzing the rise in eclectic, flexible, and constantly adaptable work-life designs [5,6].

However, this does not change the fact that “our brains are composed of billions of neurons connected to one another through myriad pathways, [and that] changing basic patterns of thought, feeling, and action requires that billions of new connections be formed. Such a process must be fed by constant experiential input and is therefore inevitably gradual [7].” Therefore, frequently experiencing sudden and radical changes is bound to challenge cognitive processes and resulting psychological states. Whether this results in more self-fulfillment or stress depends on how well those challenges are integrated into an intrinsic drive for growth and autonomy or translated into extrinsic, overloading pressure [8–10], which in turn is contingent upon the individual’s psychological safety, resilience, and life circumstances [see 11,12].

As “technology is often seen as a tool for social and economic development that is supposed to improve people’s lives, meet human needs, and achieve human goals” [13], a comprehensive and in-depth understanding of its impacts on work designs and associated psychological wellbeing is essential. This is particularly relevant with respect to work in which a fixed administrative attachment to an employer is not given, thus self-fulfilling autonomy and/or overwhelming insecurity may surface. This research therefore aims to explore and uncover the intersections between non-attachmental work designs and mental health & wellbeing as portrayed in scientific studies published in the past decade, providing a blueprint of the status-quo of the scholarly discourse and a starting point to guide specialized and comparative research as well as policy and strategic decisions in this rapidly changing era.

We approach this through a comprehensive systematic literature review using topic modeling, an automated, unsupervised, machine learning technique that enables organizing, categorizing, summarizing, and recognizing patterns in large collections of discrete text data [14,15]. The results delineate the landscape of research at the junction of non-attachmental work and psychological health, uncovering key classifications and major patterns on the prominence and evolution of emerging topics. The following sections detail the theoretical logic behind this research, before moving on to the research design and methodological details and concluding with the findings, discussion of literature, and implications for society at large.

## The rise of *no-strings-attached* work designs

At the dawn of the 20^th^ century, the emergence of the *standard employment relationship*, defined as a “stable, open-ended, and direct arrangement between a dependent, full-time employee and their unitary employer” [16], was seen as a milestone for the promotion of workers’ rights and wellbeing. This type of work was seen as a “stable, socially protected, dependent, full-time job, the basic conditions of which […] are regulated […] by collective agreement or by labor and/or social security law” [17], with “a long-term attachment to a single employer and accompanying wage and benefit expectations” [18]. This involves work relationships that are primarily characterized by the subordination of the employee to an employer who maintains control and hierarchical power over the relationship, as well as bilaterality, obligation mutuality, salary payment, and the economic dependency of the employee on this relationship as main income source [16,18].

Though employment stability began to decline in the 1980s [3,19], it has significantly accelerated “since the mid-2010s, [as] automation has replaced many repetitive error-prone administrative tasks such as processing legal documents, directing service queries and employee selection screening” while it “historically […] replaced more routine, physically demanding, dangerous, or repetitive work in industries such as manufacturing, with little impact on professional and managerial occupations” [3]. This was accompanied by the advent of the *platform-mediated gig economy* with the founding of companies such as Uber and Airbnb, both of which are now just over a decade old [20], and social media apps such as Instagram and TikTok and the associated *influencer economy* [21]. Another contributing factor is the introduction of touchscreen smartphones from around 2010 [22]. Which means, finding work at the click of a bottom has become easier than ever, with upward patterns even in the world’s most underprivileged contexts [23].

Indeed, the current world of work looks even ‘newer’ than that envisioned by Frithjof Bergmann when he first introduced the term *new work* [24], with a digital startup culture, flexible and horizontal relationships, flatter leadership styles, and entirely new economies [25,26]. New and modern work has come to refer “to a wide range of practices placed on a continuum of work flexibilization and diversification, from remote work to collaborative entrepreneurship to digital nomadism”, characterized by spatiotemporal flexibility, changing relationships between individuals and organizations, and changes in power and control structures [27]. According to Barley et al. [28], the key ways in which the nature of work is changing is the “demise of […] jobs associated with the bureaucratic employment contract in which employees exchanged labor and loyalty for security” and the emergence of “forms of employment tied to the completion of a specific task and, hence, of relatively short duration”. These have been described as *alternative work arrangements* in which there is flexibility in the scheduling of work, location in which it is accomplished, or/and employment relationship [29]. The term *nonstandard work* has also been coined, describing that which deviates from working fixed hours at a particular employer’s location and under their control with the expectation of long careers, involving a lack of temporal, physical, and/or administrative attachment between workers and the organization [30].

**Fig 1:**
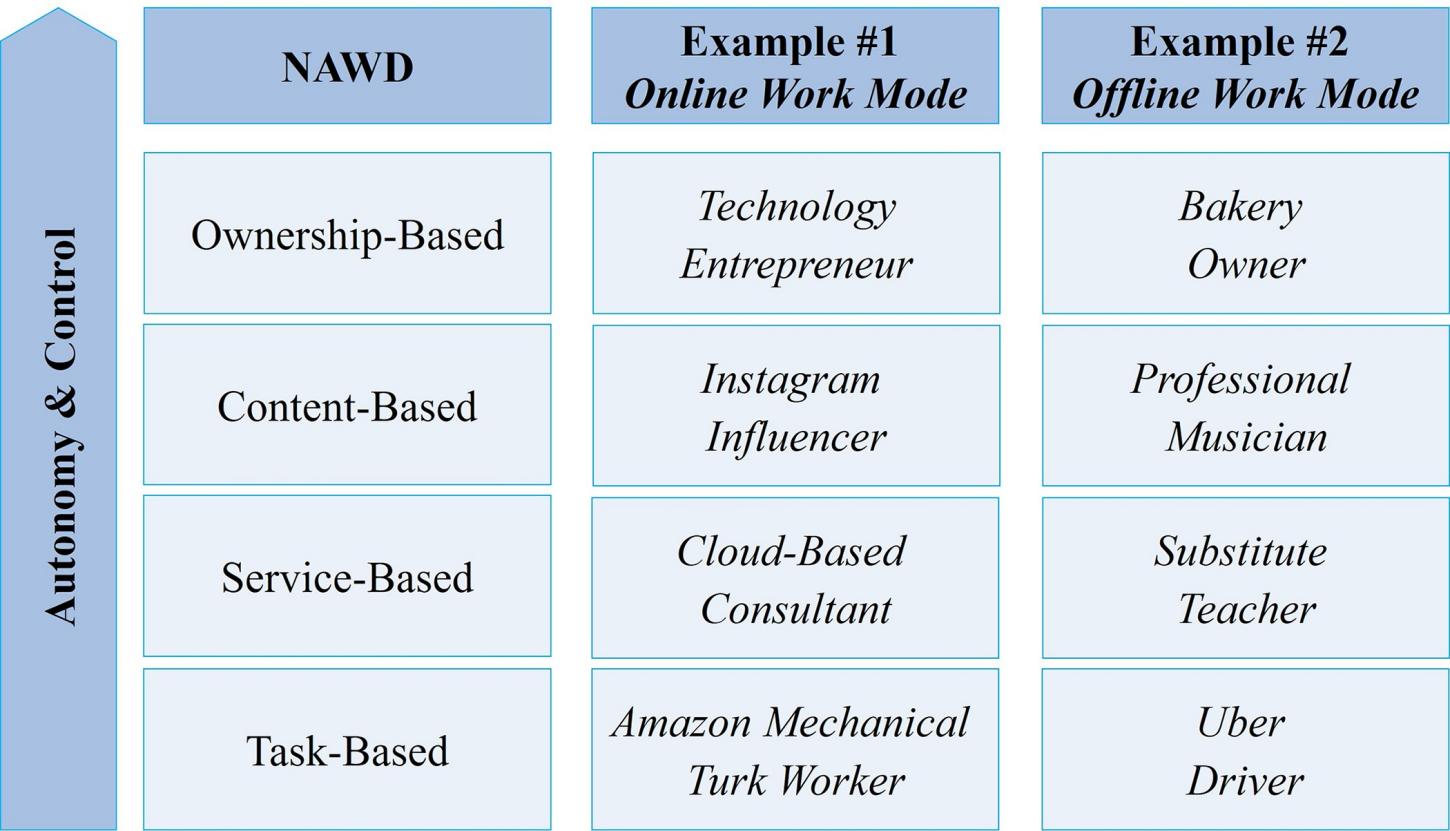
A classification of non-attachmental work designs.

This emphasizes the constant mutation in the employment relationship as the primary driver of today’s evolving no-strings-attached work culture [2,26,27,29,31]. This particularly refers to types of work in which individuals may gain income without being bound by a fixed administrative attachment to a specific employer, often with high levels of autonomy and proactivity and facilitated by modern technology, which is the focus of this paper. Those types of work will be termed ***non-attachmental work designs (NAWDs)*** for the rest of this paper, and they encompass a variety of modes and structures. In extension of the works of Spreitzer et al. [29], Vallas & Schor [20], Duggan et al. [32], Cropanzano et al. [33], Kolade & Owoseni [2], and Parker & Grote [5], we envision four different work designs that fit within the NAWD definition (see Fig 1).

The first NAWD is one where the worker aims to be paid for the creation, production, and/or sales of products or services as an owner of an organization, encompassing the likes of entrepreneurs and self-employed business owners. This kind of *ownership-based work* has been gaining pace with a global increase in rates of business ownership [34] and the emergence of new forms of technology-enabled business models [26]. Secondly, *content-based work* implies that individuals aim to gain income through content creation, often facilitated by digital platforms, such as social media influencers, vloggers, podcasters, and journalists. A third type of NAWD is *service-based work*, where an individual is paid for providing specific services for a given amount of time, which could be in affiliation with a specific project [35], and usually within the framework of a time-based contract. This encompasses temporary workers, cloud-based consultants, and freelancers such as programmers and educators. Lastly, *task-based work* is that in which a person is paid for performing specific short-term assignments, including but not limited to food delivery, babysitting, apartment and ridesharing, and maintenance and construction; a type of NAWD that has particularly boomed over the past decade with the advent of the platform-mediated gig economy. While all of those NAWDs have in principle existed before digitalization, their modern-day configuration, form, scope, adoption, and impacts are inseparable from the digital revolution. From ownership- to content- to service- to task-based work, there is a decrease in the level of worker autonomy and control with regards to shaping the work itself and designing related activities and themes. Psychological considerations are discussed in the following section.

## Implications for psychological health

Although various institutions and societies still associate the nine-to-five with safety and prosperity owing to their historical connection to workers’ rights, liberation, and social security protections, standard employment relationships may be seen differently by today’s workers, particularly younger generations [36]. Indeed, standard employment was initially designed for a highly capitalist, hierarchical, masculine, obedient, and analog workforce [37–39]. However, today’s latest workforce joiners are *digital natives*, with smart phones and internet connectivity being part of their lives since birth [40,41], and tend to be more individualistic [41], less religious [42] and therefore more inclined to reject hierarchy and dominance [43,44], more engaged in political and social activism [40], and more sustainability-oriented and conscious of environmental issues [45].

Indeed, the coupling of those sociocultural changes and access to digital tools has accelerated the transition away from traditional work structures and towards those driven by autonomy, proactivity, adaptability, personal values, and self-development [46,47]. This may promote higher levels of intrinsic motivation, self-realization, meaningfulness, and self-fulfillment [8,48], which translates to elevated wellbeing and psychological empowerment [49,50]. NAWDs may also open doors for income generation to humans from all walks of life, even those in geographically isolated and institutionally fragile locations [51,52], enhancing psychological safety and security. The flexibility arising from digitally enabled work may also promote work-life balance and family satisfaction [53].

Meanwhile, “tasks that people are more likely to do in future work will require high-level cognitive and emotional skills that are more likely to be developed, used, and sustained when underpinned by self-determined motivation” [3]. This means that those who may not be privileged enough to pursue or possess the psychological stability and emotional support needed to thrive in this new world of work may indeed be left to struggle [e.g. 54]. This mostly applies to those who find themselves at the base layers of Maslow’s pyramid [55], striving to fulfill survivalist, physiological needs such as food and shelter rather than self-actualization. In the words of Kößler et al. [56], “satisfying self-actualization needs might not be a priority for people who are restricted in their job choice and who are in the first place preoccupied with providing for the livelihood of themselves and their family”. Hence, NAWDs may promote psychological stress and anxiety [4,39,46], particularly for risk-averse individuals who have more to lose.

Additionally, work that is digitally mediated may promote the need for constant availability and a decline in worker self-control, resilience, and problem-solving capabilities [13], further compromising psychological health. The need to constantly develop new skills and reinvent one’s work identity may lead to stress and overchallenge, lack of purpose and commitment, and a constant feeling of “I am not good enough” [46,47]. Frequent transformation and adaptation may also limit workers’ “chances of reaching maturity and high-level achievements” [46], which may on the one hand relieve the pressures and moral dilemmas associated with the need to get promoted at work [57], while on the other may lead to purposelessness, amotivation, and dispensability. NAWDs may lack the sense of community and teamwork that comes with a traditional workplace [58,59], which may lead to either individual empowerment and achievement or social isolation and disenfranchisement [31].

**Fig 2:**
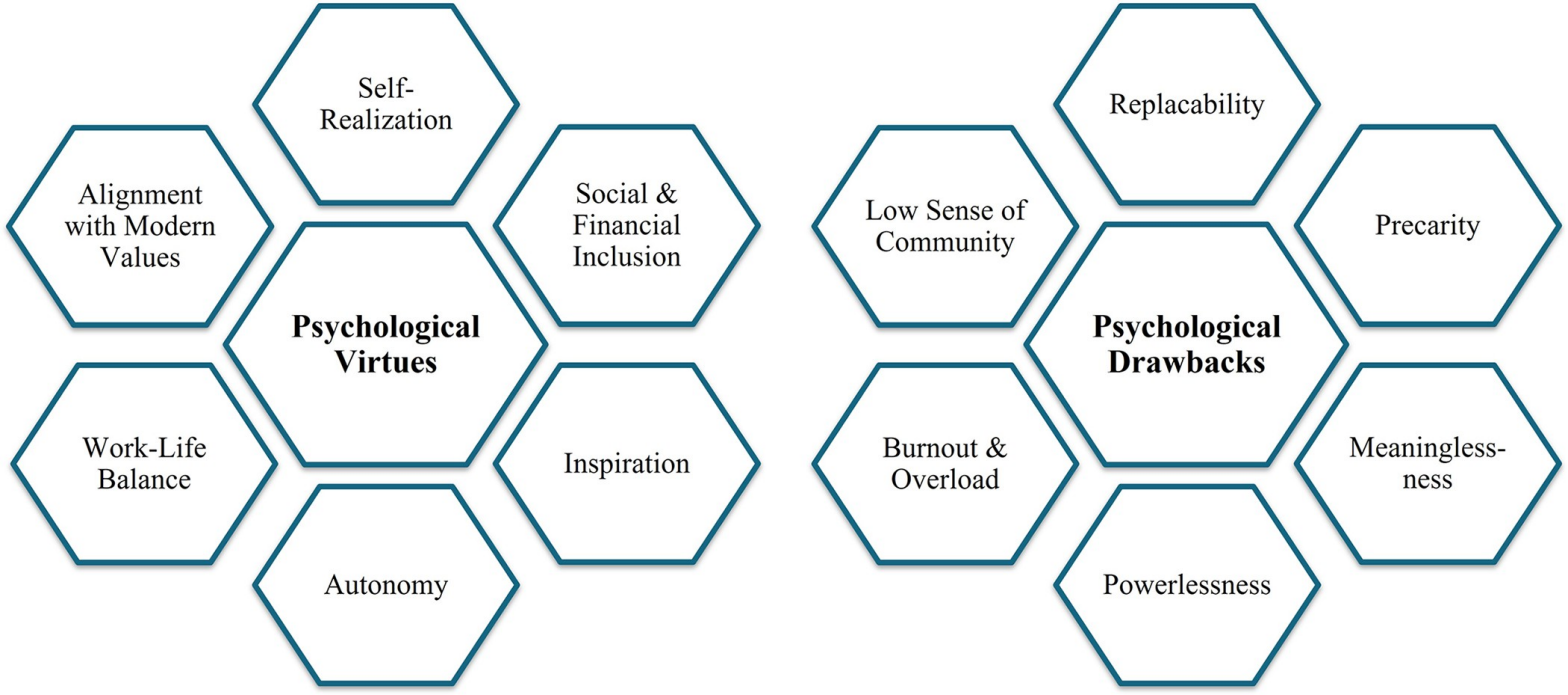
A summary of the bipolarities of the psychological impacts and considerations in association with non-attachmental work designs.

Digital technologies may therefore both increase or decrease motivational work characteristics, satisfaction, and social wellbeing [3,60,61], and this uncertain and everchanging world of work may evoke both positive and negative psychological states (see Fig 2). One worker might perceive this uncertainty as interesting, exciting, a learning opportunity, or socially encouraged, while another perceives it as rather stressful, precarious, or overwhelming [51,62,63]. Work-associated freedom, sense of meaning, security, and cognitive load play a major role [64]. Therefore, each specific NAWD, with its associated opportunities for autonomy and control, may impact psychological health differently. An individual who designs their work with high levels of freedom, such as an entrepreneur or digital content creator, may enjoy more self-fulfillment and inspiration or on the other hand struggle with overwhelming responsibility and technostress. Meanwhile, a gig worker may experience more work-life balance with a sense of little to lose, while battling feelings of replaceability, insecurity, and being confined in an *invisible cage* where the ability to thrive, grow, and succeed falls increasingly beyond their control [65].

## Analysis

Given the grave importance and relevance of the topic at hand, this research aims to provide a blueprint of the status-quo of the non-attachmental work versus psychological health debate through **exploring and uncovering the intersections between NAWDs and mental health & wellbeing as portrayed in recent scientific studies**. This serves as a cornerstone on which to further build specialized and comparative research as well as inform policy decisions and societal actions. Accordingly, a review of recent literature was performed using unsupervised topic modeling [14,15]. Methodological details are provided in the next two sections.

### Database

Since this research aimed to construct an as complete as possible blueprint of modern literature at the intersection of NAWDs & psychological health, which consists of a wide variety of specific work arrangements as well as a wealth of psychiatric issues, mental health conditions, and wellbeing-related states, a highly comprehensive and carefully selected list of keywords and search terms was curated. With respect to NAWDs, a list of 52 search terms was constructed based on a review of the works of Aroles et al. [27], Ashford et al. [30], Barley et al. [28], Spreitzer et al. [29], Vallas & Schor [20], and Sundararajan [66] followed by confirmation with six external organizational researchers from two German research institutions. As for search terms related to psychological health, several peer-reviewed papers as well as the formal publications of accredited health institutions have been examined [67–74], resulting in a list of 83 search terms that was then confirmed with two external psychologists from one German and one Norwegian research institution. The full lists of search terms are available in the as supporting information (S1 Appendix).

The search terms were used to construct search queries to extract literature from five different databases; EBSCO Business Source Premier; Web of Science Core Collection; Medline; APA PsycInfo; Psychology and Behavioral Sciences Collection. This was done to ensure the comprehensiveness and robustness of literature choice across a variety of disciplines including psychology, management, the social sciences, and medicine. The search criteria was limited to peer-reviewed papers published between January 1^st^ 2012 and March 31^st^ 2022 in the English language. The results were exported to Zotero reference management software for formatting and duplicates removal, before exporting those 3511 publications as a single database to Microsoft Excel for further processing.

This was followed by several rounds of processing which included formatting and duplicates removal, as well as the manual exclusion of articles that were out of scope through examination of their titles and abstracts. The latter was often the result of search term ambiguity, where certain words possess multiple meanings. For instance, “bipolar” could refer to bipolar disorder, a psychiatric condition, or an attribute of magnets and metal electrodes, and “founder” could either refer to a startup entrepreneur or a genetic mutation. In some cases, an examination of a full-text article may have confirmed that a clear aspect of either mental health or non-attachmental work was indeed absent, leading to further exclusions. These noise-reducing processing steps [75] resulted in a final database of 1094 papers, all of which were downloaded as full-text PDFs and converted into text files, which were then cleaned by the removal of references, headers & footers, and author names in preparation for topic modeling.

### Topic modeling

To analyze the content of the scientific papers, we chose an unsupervised machine-learning approach: the Latent Dirichlet Allocation (LDA) topic modeling algorithm [14,76]. From a technical point of view, “topic modeling algorithms are statistical methods that analyze the words of the original texts to discover the themes that run through them, how those themes are connected to each other, and how they change over time” [76]. Thus, each topic consists of a series of words that frequently occur together. Topic modeling has become a well-established method for the analysis of public narratives, scientific debates, and academic literature reviews [e.g. 77–80], also with respect to mental health research [e.g. 81,82]. Its usefulness has been shown when analyzing the development of a research domain [83,84]. As topic models are based on probability distributions, each document is characterized by a number of topics with specific weights, which allows analyzing topic frequency over time, for example “hot” and “cold” topics [85,86].

For a topic modeling analysis, several preprocessing steps are necessary [77,87,88]. First, the text has to be made ‘machine readable’. For this purpose, we used part-of-speech-tagging and lemmatizing [89–91] to make the texts easier for the algorithm to analyze. Part-of-speech-tagging is a classification process that aims to assign a particular part of speech to each word in the corpus. This allows researchers to filter the corpus for ‘meaningful’ words like nouns and adjectives and to exclude, for example, articles or relative pronouns. Second, we used lemmatizing to group the inflected forms of a word (e.g., *supporting*, *supported*, and *supportive* are transformed to their base form: *support*). These procedures increase the speed of the modeling algorithm as well as the quality of the findings [88] through preventing the emergence of topics consisting solely of different declensions of the same word [91]. In addition, the text was tokenized (divided into sets of meaningful pieces) and converted to lower case. Numbers and punctuation marks were also removed as well as stop words, namely extremely common words like auxiliaries. Those steps and a quantified representation of the resulting dataset are shown in Table 1.

**Table 1:** A summary of the topic modeling steps reported in accordance with Hickman et al.’s [88] best practices for reporting text mining procedures.

Reporting	Information
Source of data	1092 academic papers.
Pre-processing	Tokenization; lowercase conversation; stop word removal; POS tagging and removal of meaningless words; lemmatization.
Characteristics of the final data set available for topic modeling	Vocabulary size: 14,482. Number of words: 3,296,681.
Used software and version	Python 3.X, lda 1.0.5 package available here and tmtoolkit developed by Markus Konrad available here.
Model settings	Beta = 0.01, alpha = 50/K, where K = number of topics.

**Fig 3:**
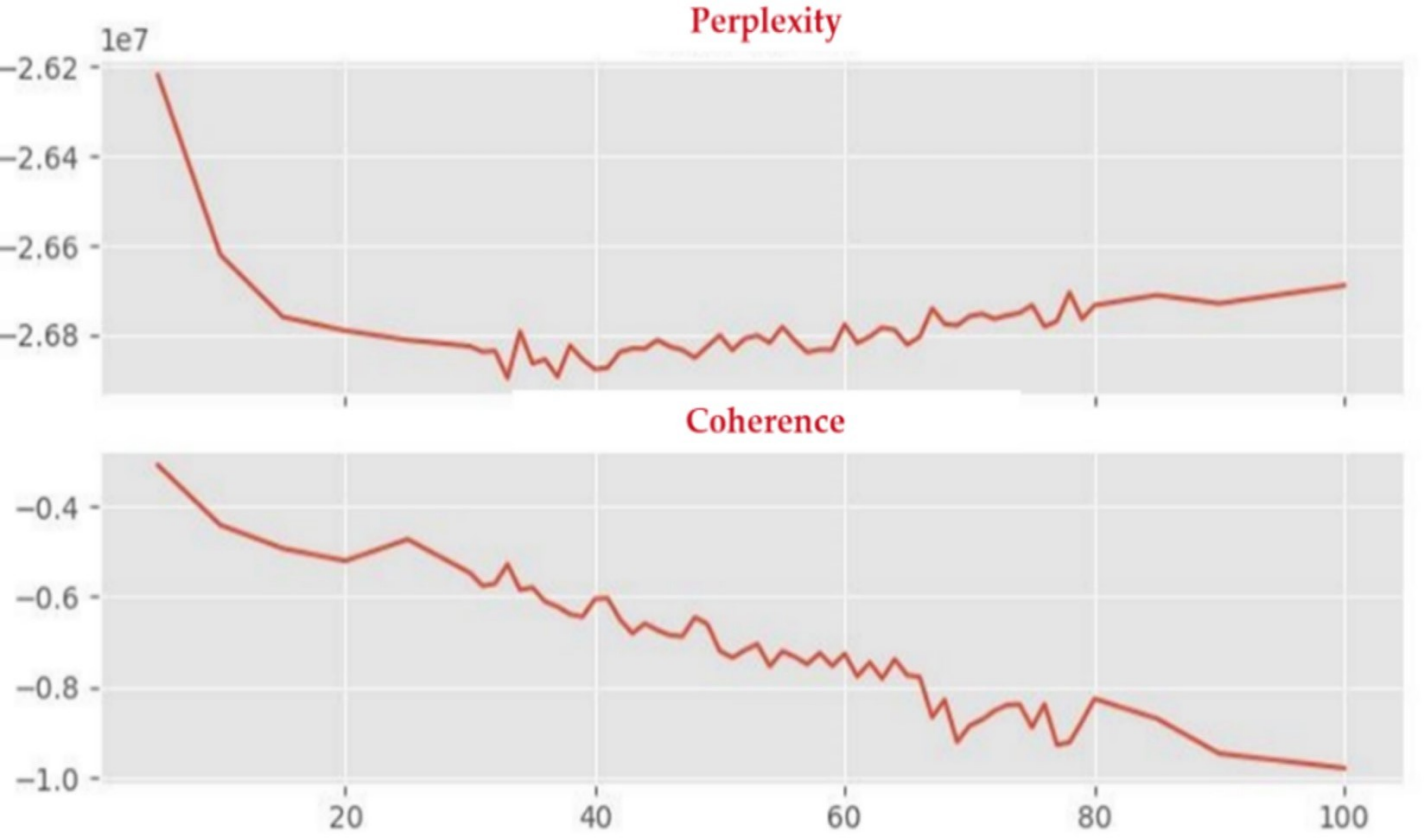
The perplexity and coherence plots analyzed to pinpoint optimal model size.

Following text pre-processing, standard software was used to compute a range of 60 models containing from five to 100 topics [90,92]. This was done using python 3.X and the tmtoolkit, and the specific code is available by the authors upon request. Standard metrics where then used to pinpoint the models consisting of the optimal number of topics, aiming for those with low perplexity and high coherence [86,87]; in other words minimizing model fit uncertainty and maximizing the results’ interpretability. Several models were found to have a good fit with regards to the combination of both those criteria, namely those consisting of 33, 36, 41, 47, 65, 80 topics, as indicated by Fig 3.

To select the optimal model, those six models were qualitatively analyzed and manually compared. In line with DiMaggio et al. [89], each of the models was evaluated in order to find the one with the best fit in terms of interpretability or *logic of fit* [90]. In other words, the most semantically meaningful model was chosen, that which best portrays the complexity and diversity of the research landscape at the intersection of NAWDs and mental health in a fine-grained manner while minimizing jargon and text data noise. Smaller models were found to be highly general and less differentiated in comparison with the 80-topic-model, which was ultimately selected as the one offering the best fit in terms of perplexity, coherence, qualitative complexity, and interpretability.

### Data coding

An inductive approach guided by Gioia et al. [93] was used to interpret the final model. Each of the 80 topics was portrayed as a distribution of words that were listed in an Excel table and visualized in network form with the aid of the LDAVis tool to aid with the topic structuring and categorization [94] as well as in word clouds. Coding the topics to identify the logics was a two-part process. In part one of the process, inductive and open coding was performed, where the individual words and the top-loading scientific articles associated with each topic were thoroughly analyzed [93]. The loadings were computed by the LDA algorithm. Those considered top-loading documents were those with a correlation coefficient of 10% or higher to the corresponding topic [83,95].

Accordingly, each topic was given a brief title, or first-order code, which was discussed internally to ensure representativeness and suitability. The first-order codes were first given to each topic by the lead author after having thoroughly read the abstracts of the majority of the top-loading documents pertaining to the topic to understand topic content. In cases where a clear connection between documents within a topic was not immediately visible, the topic was flagged for internal discussion. The process was then repeated by the second author of the study, albeit with more focus on analyzing the top 20 most associated words with each topic. Topics were also flagged for internal discussion if a clear overarching theme was not immediately detectable. This was followed by a comparison of first-order codes generated by both authors, with a particular zoom-in on the ambiguous topics identified by both. There was clear consensus on first-order codes on the vast majority of the topics, and in cases where the authors had different perspectives, both the abstracts of top-loading documents and most frequently occurring words were reanalyzed jointly by both authors and discussions were conducted until agreement was reached. Examples of those topics included #19 (Temporal Perspectives), #34 (Values & Power Dynamics), #36 (Work-from-Home Consequences), and #64 (Online Microtasking & Mental Health (Experiments)). The finalized list of first-order codes was then explained to and approved by the third author.

In part two of the process, those topics were grouped into more general second-order themes, or overarching categories, based on the NAWDs they primarily represent (*ownership-based*, *content-based*, *service-based*, and *task-based* work designs). This was based on an estimation of the work design that is most prevalently discussed in the highly loading papers associated with a topic. In cases where several work designs are discussed in the papers pertaining to a topic or at least two work designs are represented fairly equally, the topic was labeled *cross-cutting*. Additionally, a sixth second-order code emerged and was labeled *methods* as it corresponded to topics consisting of words and articles that do not correspond to a specific aspect of mental health or NAWDs, rather those representing articles that share a specific methodological design.

In addition to topic labeling, each topic was also assigned a color representing its “coldness” or “hotness”. Cold topics are those where research interest has declined over the past decade (blue-colored), while hot topics are those exhibiting increasing research attention in the same time period (red-colored). This was done through fitting linear models for each topic over this time-period [96], which involved a comparison of the relative occurrence of a topic at the beginning and at the end of the study period. The complete results are detailed as supporting information (S2 Appendix) and in Figs 9 and 10.

## Findings

### Dataset descriptive patterns

**Fig 4:**
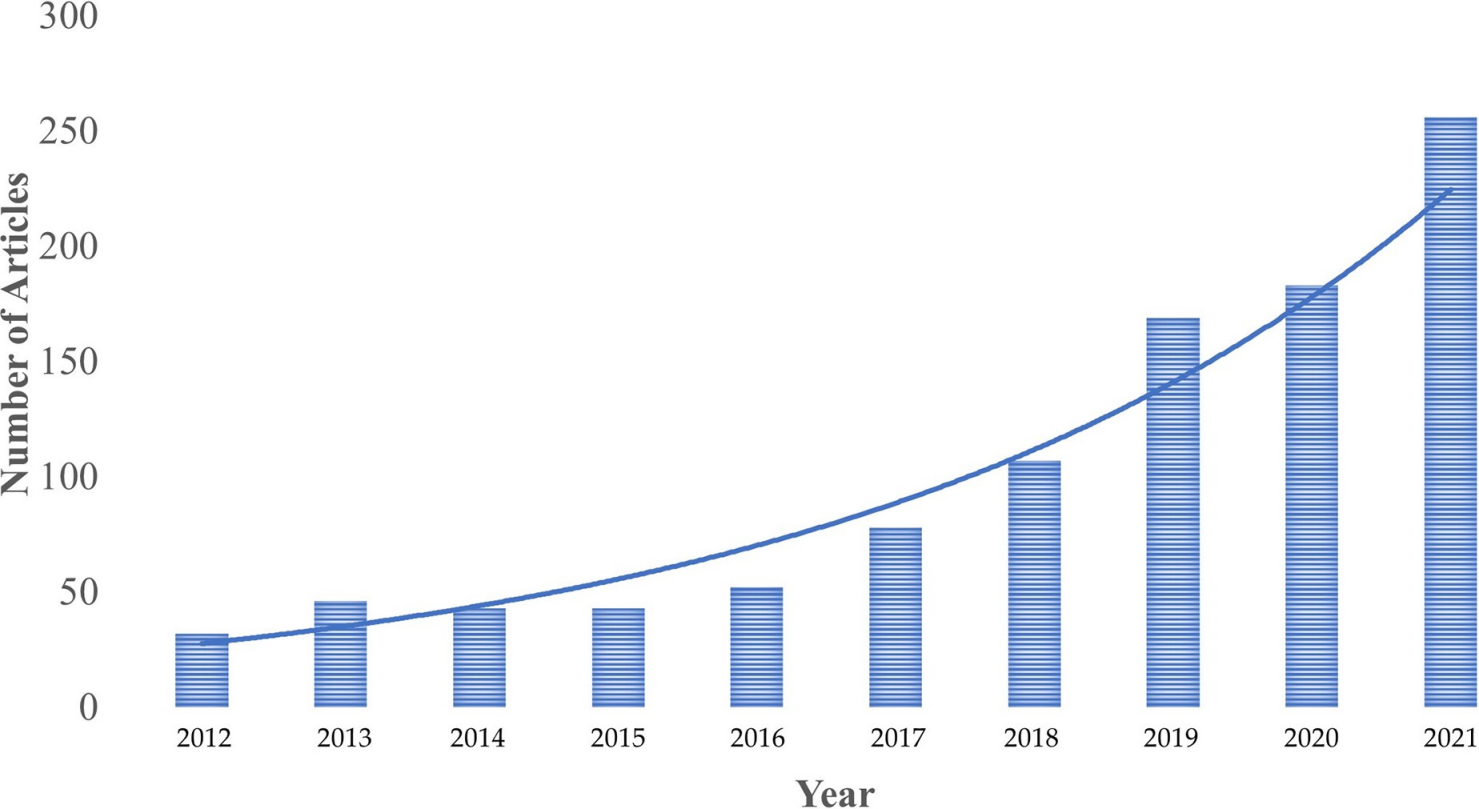
A bar chart indicating the exponential upward trend in scholarly interest at the intersection of NAWDs and mental health as indicated by the yearly number of published scientific articles. The year 2022 has been omitted from this chart as only data from the first months of the year is available.

Clear trends emerge from our dataset. Generally, there is an exponential increase in scholarly interest at the intersection of various aspects NAWDs and mental health and wellbeing over the past decade (see Fig 4). With regards to publication outlets, there is a wide diversity in terms of journals from which the dataset is formed, across the disciplines of psychology, technology, management, sustainability studies, sociology, and public health. However, one can notice a predominance of entrepreneurship journals, even though the term *entrepreneurship* was only one of 52 NAWD-related search terms used for the literature search (Table 2).

**Table 2:** The top 25 journals from which the dataset of 1094 scientific articles originates.

Journal Name	Number of Articles
Frontiers in Psychology	40
Small Business Economics	39
Entrepreneurship: Theory & Practice	33
Journal of Business Venturing	33
Journal of Business Research	25
Sustainability	25
International Journal of Entrepreneurial Behavior & Research	23
International Journal of Environmental Research & Public Health	20
International Entrepreneurship & Management Journal	16
Entrepreneurship Research Journal	13
Journal of Entrepreneurship In Emerging Economies	11
Journal of Happiness Studies	11
Journal of Small Business Management	11
Academy of Management Perspectives	10
International Small Business Journal: Researching Entrepreneurship	9
Journal of Enterprising Communities	9
Journal of Small Business & Entrepreneurship	8
Computers in Human Behavior	7
Journal of Business Ethics	7
Applied Psychology: An International Review	6
Applied Research in Quality of Life	6
BMC Public Health	6
Economic & Industrial Democracy	6
Entrepreneurship & Regional Development	6
International Journal Of Hospitality Management	6

**Fig 5:**
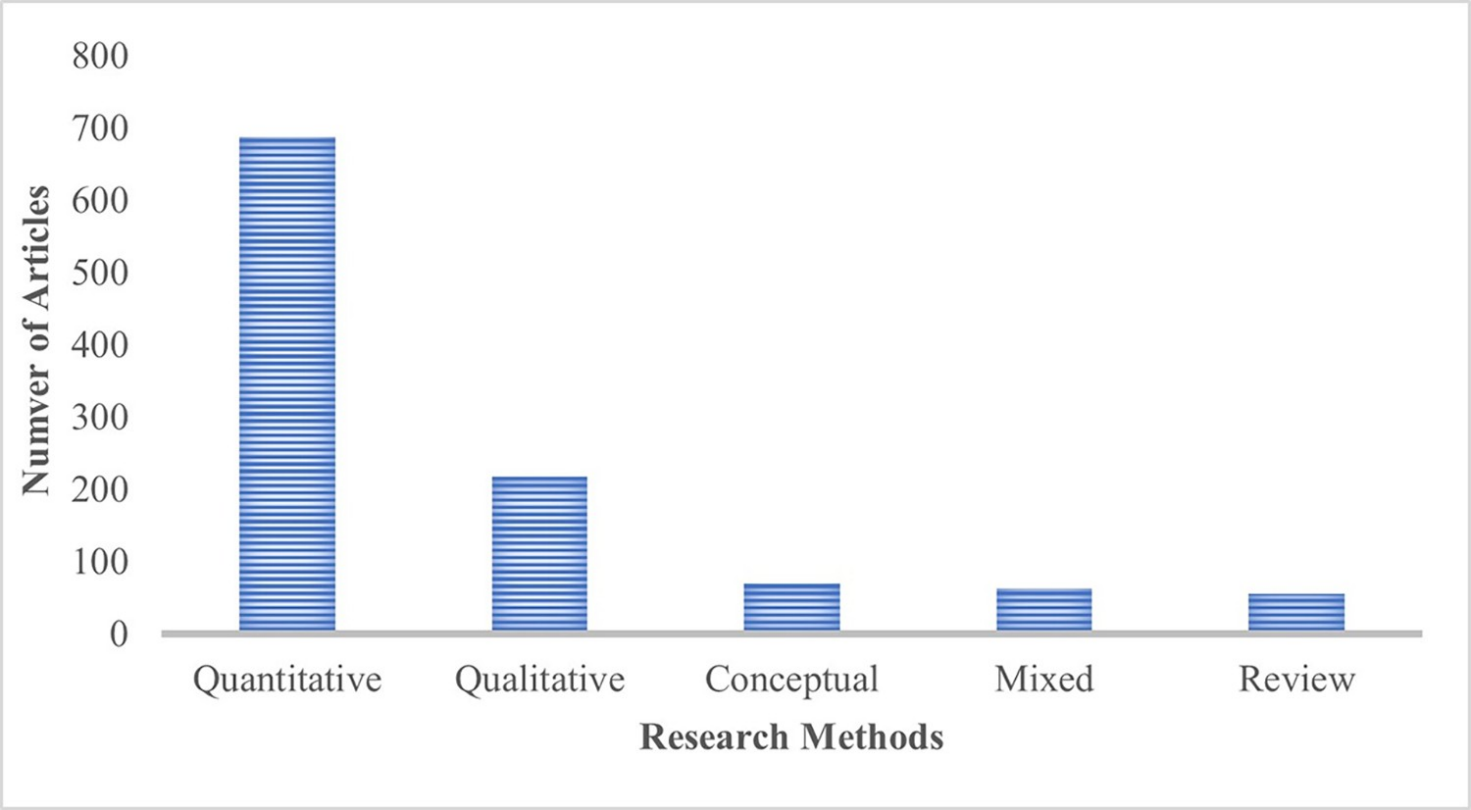
A bar chart indicating the research designs and methods associated with the scientific articles at the intersection of NAWDs and mental health and wellbeing in our dataset.

**Fig 6:**
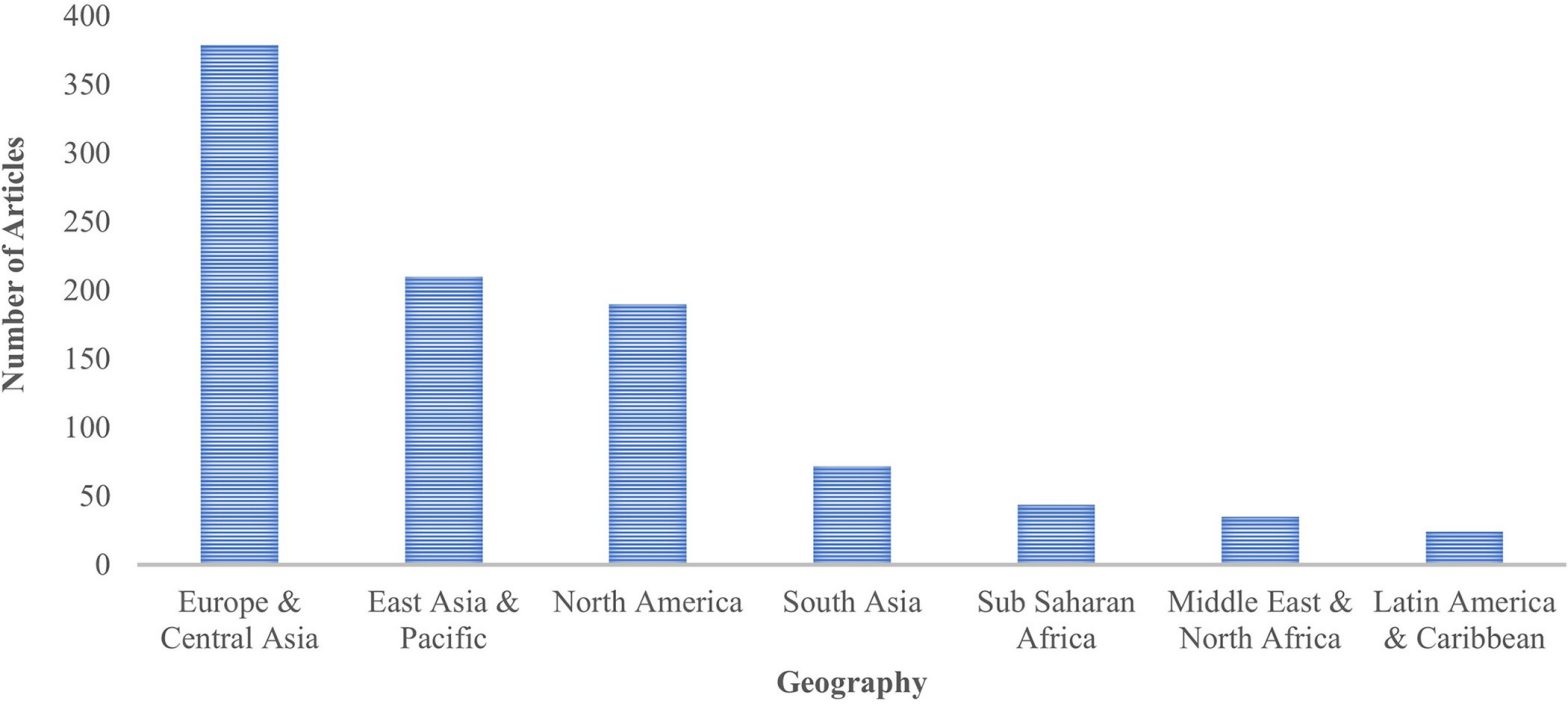
A bar chart indicating the number of published empirically driven scientific articles in each geographic region at the intersection of NAWDs and mental health and wellbeing.

With regards to the research methods employed across the articles in the dataset (Fig 5), we find that quantitative papers constitute a clear majority (63%), followed by qualitative empirical papers (20%). Generally, 927 out of the 1094 scientific articles in the dataset are empirical research papers (which includes articles combining mixed methods) with data collected from up to two specific geographic regions with respect to the World Bank geography classification [97]. Over a third of all those articles involve research in Europe & Central Asia, while least-researched regions are Sub Saharan Africa, the Middle East & North Africa, and Latin American & the Caribbean, respectively (see Fig 6).

### Emerging topics

**Fig 7:**
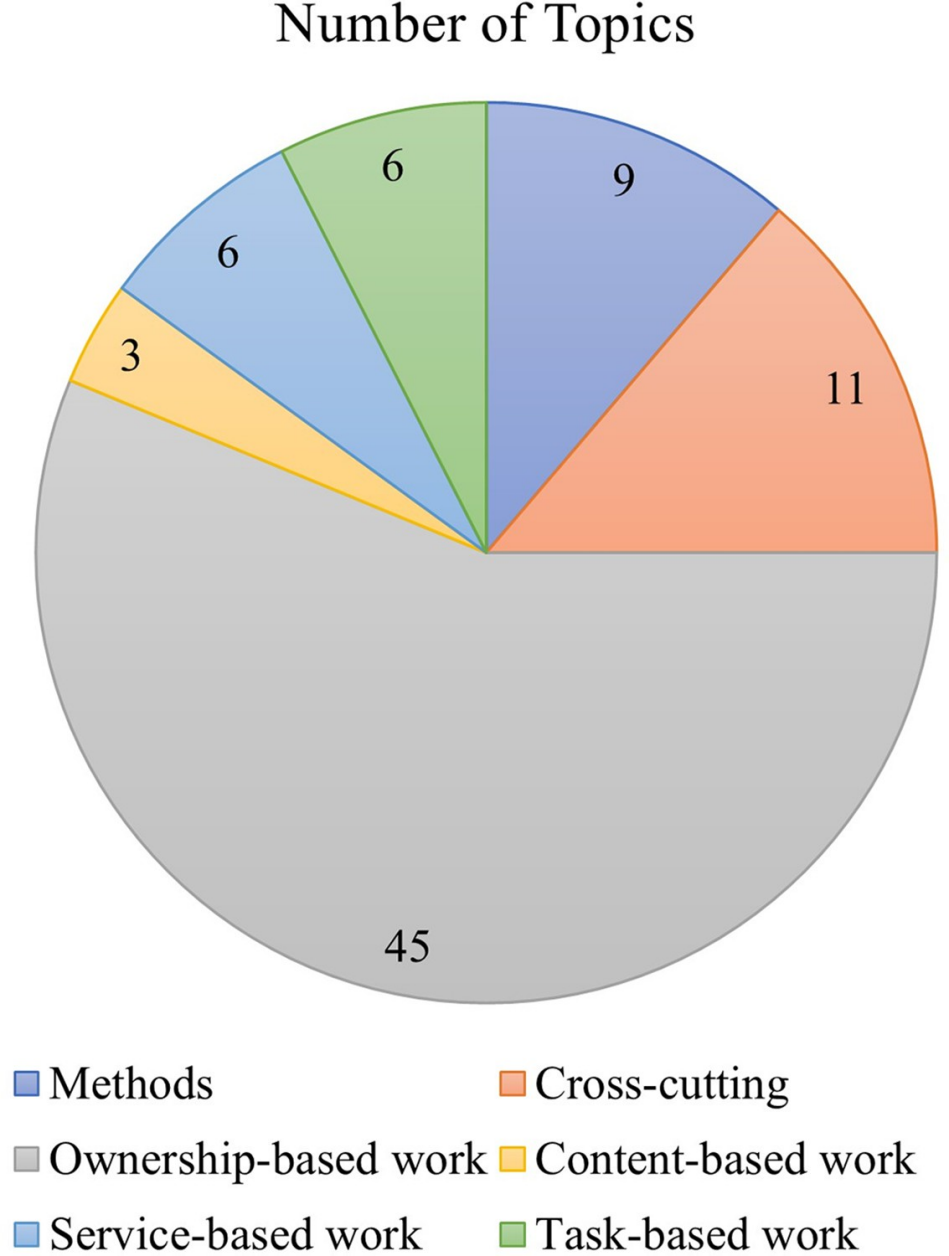
(left): A pie chart indicating the number of topics associated with each overarching category.

**Fig 8:**
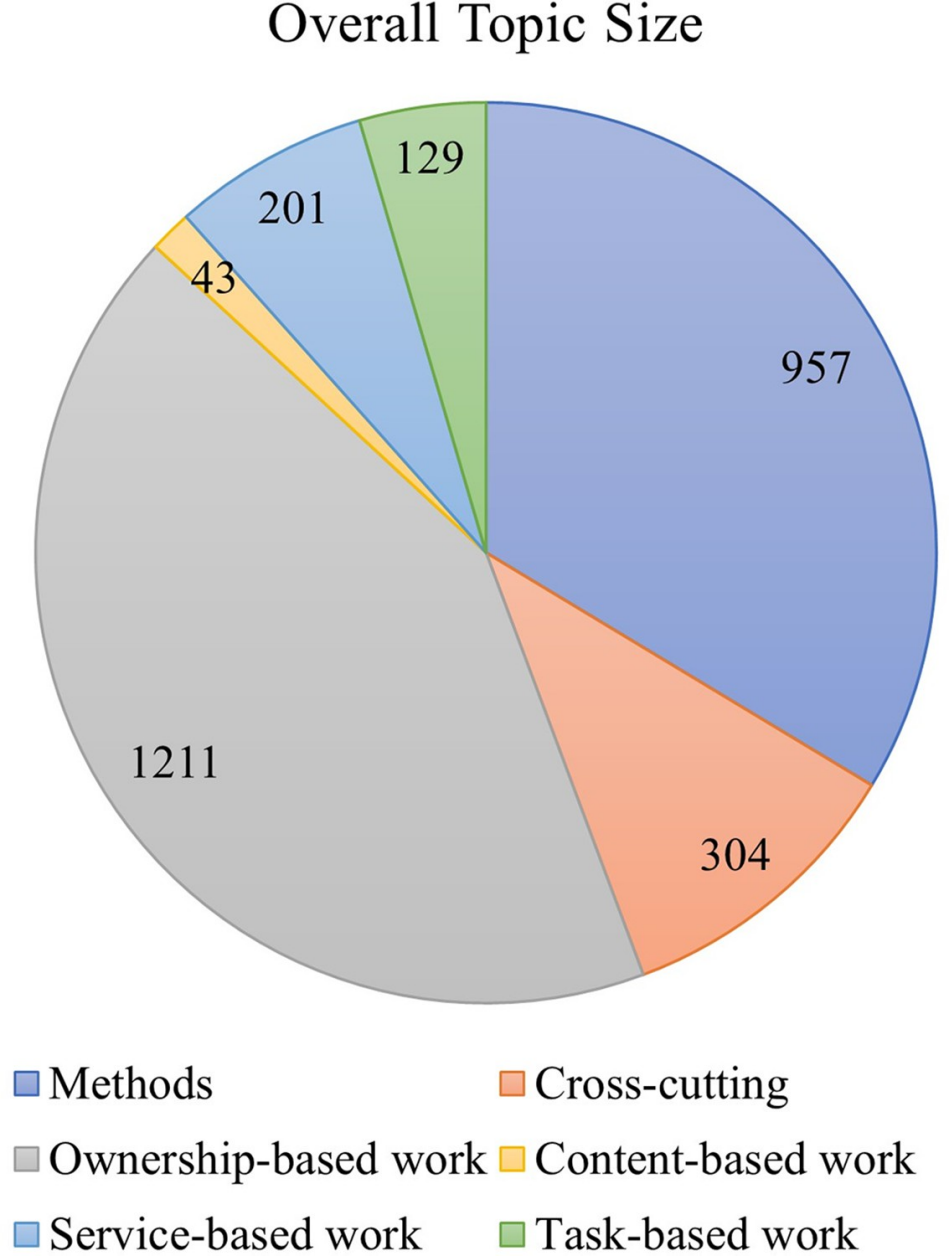
(right): A pie chart indicating the overall topic size for each overarching category, calculated as the total number of articles with a loading of 10% or higher that are associated with all topics per category. Since an article may be associated with several topics, the total article number per overarching category may be higher than the total of 1094.

The division of the 80 topics into the six overarching categories yielded the following; nine are methods topics, 11 are cross-cutting topics each of which relates to several NAWDs, 45 relate to ownership-based work, three relate to content-based work, and service-based work and task-based work each consists of six topics (see Fig 7). The overall topic sizes in each overarching category, calculated as the number of articles with a loading of 10% or higher that are associated with all the topics that belong to this overarching category, were also calculated and indicated in Fig 8. The following sections describe the topic modeling findings in each of those overarching categories.

## The methods cluster

A closer examination of the *methods* topic cluster, which consists of eight topics, reveals a predominance of quantitative methods, in alignment with the dataset characteristics, albeit with a downward trend with regards to employing macro-level panel data (indicated by the “coldness” of topic #31). Meanwhile, an upward trend in the use of longitudinal study designs and temporal data is indicated by the “hotness” of topic #19. This signals a shift of research interest to the human (worker) level rather than the macroeconomic context, while taking longer term development into consideration. Topics #1, #29, #31, #38, and #76, all of which are under the *methods* category, are the five largest topics in terms of total article count across the entire dataset. This is largely due to the fact that research methods and designs are relatively standard across various research fields, hence few terms pertaining to research methods appear in a large number of articles.

## Predominant themes across all NAWDs

The remaining 71 topics can be loosely classified into four, non-mutually exclusive dimensions. The first concerns general psychological health at the level of a particular geopolitical, cultural, or societal context, for example topics #21 (healthcare System & Policy) and #48 (National Wellbeing & Happiness). The second dimension zooms in on sociodemographic aspects of psychological health, exemplified by topics #6 (Gender & Racial Biases), #61 (Old Age & Wellbeing), and #80 (Latino Day Laborers, Alcoholism, & Discrimination). The third dimension concerns general psychological health in particular organizational settings and work environments, such as topics #25 (Conflict & Satisfaction in Teams), #39 (Online Microtasking & Wellbeing), and #57 (Wellbeing in the Hospitality & Food Industries). Finally, the fourth dimension concerns particular psychological attributes, states, and traits at the personal level, namely emotions, motivations, and cognitions, with topics such as #7 (Affect), #11 (Life Satisfaction), #14 (Resilience), #40 (Emotions & Self-Expression), #44 (Personality Traits), #54 (Autonomy & Self-Fulfillment), #67 (Intrinsic Motivation), and #73 (Self-Concept). This is illustrated in Fig 10.

**Fig 9:**
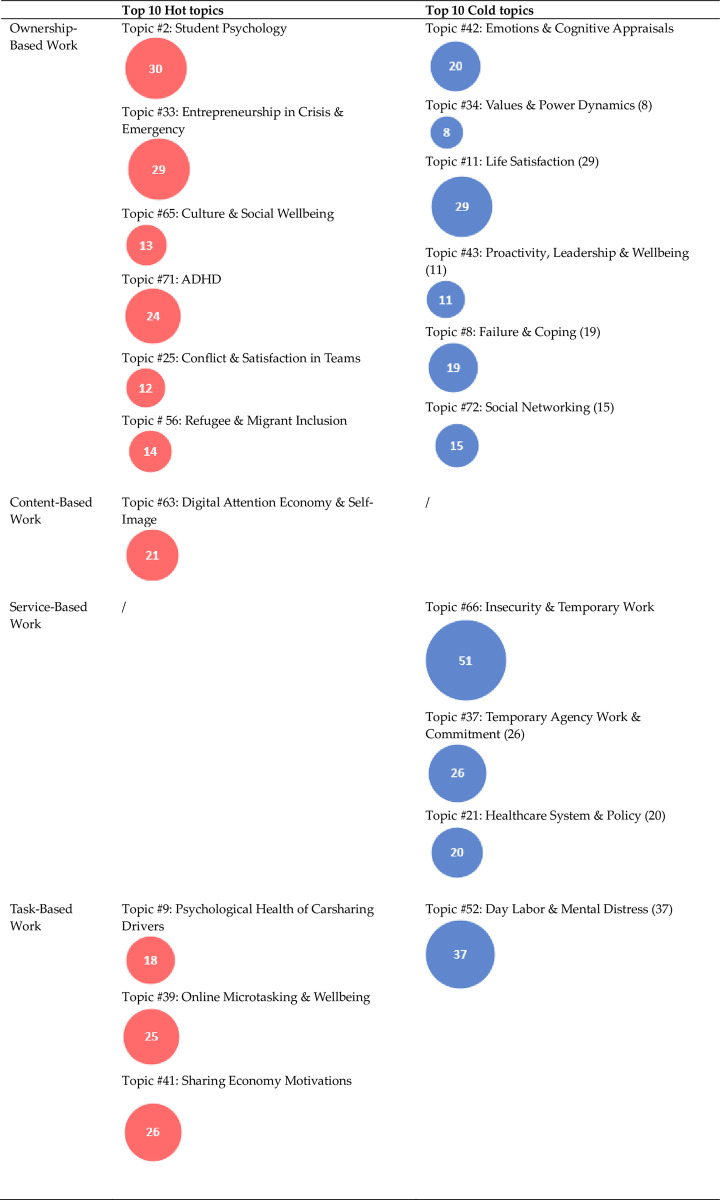
A summary of key hot and cold topics, where the numbers in the circles correspond to the total number of scientific articles with a loading of ≥10% with regards to the topic (see also S2 Appendix).

Out of the 80 topics, 11 concern psychological health themes that appear in articles corresponding to several NAWDs, hence their second-order code is labeled *cross-cutting*. These mostly pertain to the societal context, such as topics #4 (work & family) and #20 (Community Empowerment in the Global South), psychological motivation, such as topics #15 (Crowdsourcing Motivation) and #60 (Engagement & Meaning), and mental overload, such as topics #30 (Depression), and #74 (Sleep & Exhaustion). Most of those topics are either cold topics or neutral, indicating either a decrease or no markable change in research attention since 2012, with the exception of #32 (Farm Labor & Household Wellbeing). The coldest amongst the 11 topics is #36 (Work from Home Consequences), which touches upon the challenges faced by workers who had little separation between home and work life before the pandemic-related boom in home office culture, such as online language translators [e.g. 98] and Bed & Breakfast Innkeepers [99], in contrast to the hot topic #33 (Entrepreneurship in Crisis & Emergency) that largely focuses on the consequences of the COVID-19 pandemic. Fig 9 illustrates the hottest and coldest topics pertaining to each of the four NAWDs.

## Psychological health & ownership-based work

A striking 45 of those 60 topics pertain primarily to ownership-based work. The majority of those 45 topics are hot topics, indicating a general increase in research interest at the intersection of entrepreneurship and psychological health. We also observe that wellbeing aspects, rather than illbeing or mental illness, seem more common among those topics, particularly those belonging to the psychological attributes, states, and traits dimension. Indeed, no topics emerged with respect to specific diagnosable mental health conditions or illnesses except for Attention Deficit Hyperactivity Disorder (topic #71; ADHD), which also appears to be a highly hot topic, but is rather formulated positively as a promoter of ownership-based work. Topics that concern illbeing and mental health issues include #8 (Failure & Coping), #17 (Entrepreneurial Stress), and #75 (Passion vs. Obsession), none of which is however a hot topic.

**Fig 10:**
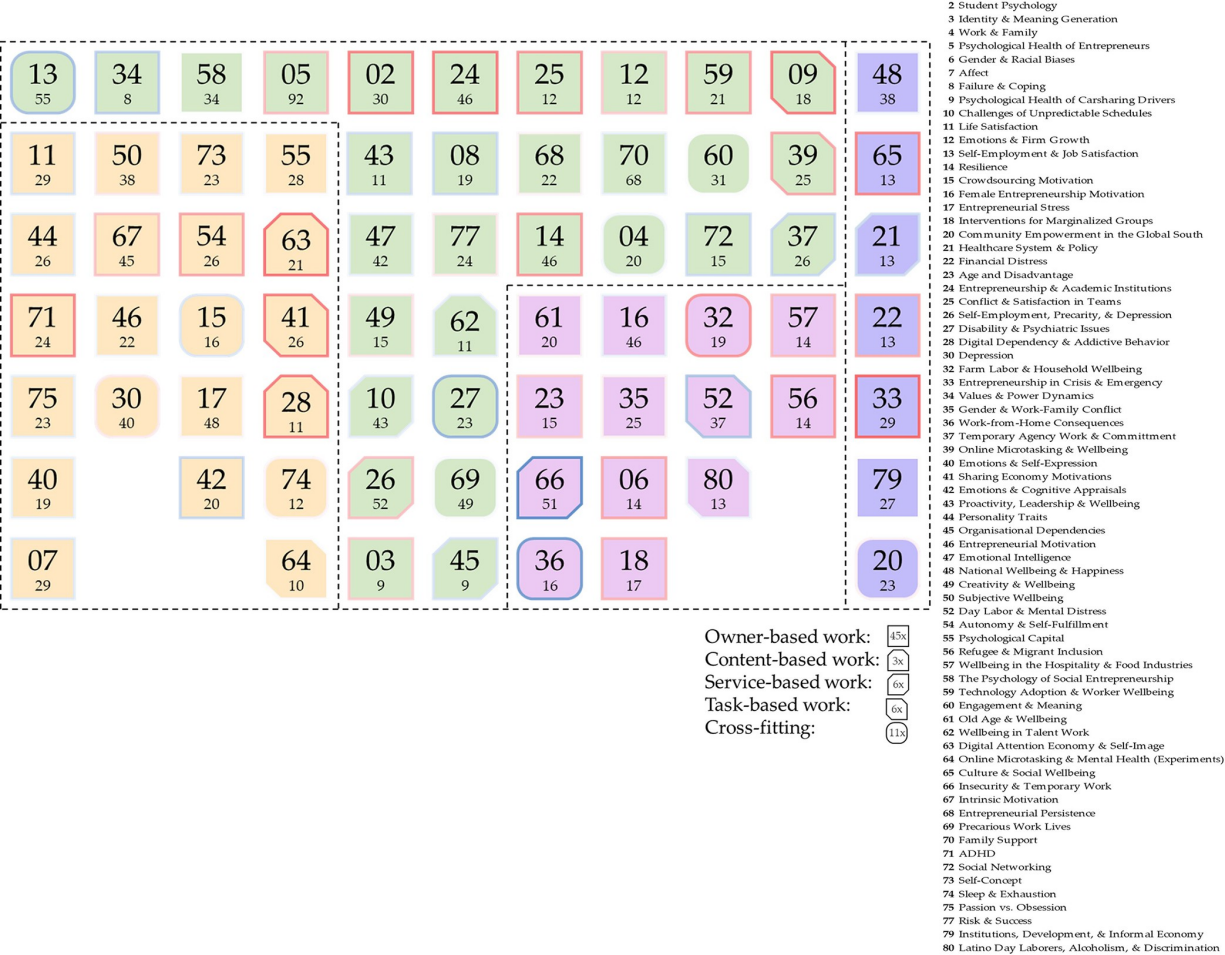
An illustration of the key patterns and characteristics of the 71 non-methods topics. Each icon represents one topic whose number is written in the center (see the legend on the right for the corresponding topic titles). The icon shape indicates the NAWD to which the topic belongs. The icon fill colors correspond to the four dimensions: purple includes topics on psychological health at the level of a particular geopolitical, cultural, or societal context; pink corresponds to topics handling sociodemographic aspects of psychological health; green topics concern general psychological health with respect to organizational settings and work environments; yellow topics zoom in on specific psychological attributes, states, and traits at the personal level (i.e. emotions, motivations, and cognitions). The smaller numbers at the bottom indicate the topic size. The border color corresponds to the hotness or coldness of the topic, mirroring the leftmost column in the table in the Supporting Information section.

Research on ownership-based work is seen on all four dimensions (Fig 10). Moreover, some sub-patterns emerge that are unique to this particular NAWD. For instance, topics on gender and family seem to be quite exclusive to ownership-based work, with exception of the cross-cutting topic #4 (Work & Family). This includes topics #16 (Female Entrepreneurship Motivation), #35 (Gender & Work-Family Conflict), and #70 (Family Support). A second observation is that only one of the 45 topics shows a clear consideration of digital technology and its impacts on ownership-based work, namely 59 (Technology Adoption & Worker Wellbeing).

## Psychological health & content-based work

In stark contrast to research on ownership-based work, only three topics primarily concern content-based work. Indeed, it appears that only limited research attention is given to the psychological health of content creators who depend on digital media to generate income, though much research targets the mental health of consumers of such content [100–102]. We observe that research on content-based work specific handles psychological states without explicit consideration of regulatory, institutional, or sociodemographic aspects.

Two of those topics, #28 (Digital Dependency & Addictive Behavior) and #63 (Digital Attention Economy & Self-Image) are hot topics where primarily issues of social media addiction and self-image are addressed amongst digital content creators. Those two topics, albeit small in size, portray the dichotomies associated with the mental health consequences of digital content-based work. This ranges from addictive behaviors in the attention economy [e.g. 103], to algorithmically perpetuated self-doubt and inequalities [e.g. 104], to body-image empowerment of underrepresented communities [e.g. 105]. Meanwhile, topic #62 (Wellbeing in Talent Work) is a slightly cold topic that concerns psychological health and non-digital content creation, particularly amongst freelance artists.

## Psychological health & service-based work

This six-topic cluster is comprised almost entirely of cold topics (5/6 topics) and mostly concerns temporary non-digital project-based work. Unlike ownership-based work, these topics highlight the negative psychological aspects of service-based work, as seen in topic #10 (Challenges of Unpredictable Schedules), #26 (Self-Employment, Precarity, & Depression), and #66 (Insecurity & Temporary Work). The remaining three topics concern the nuances of institutional arrangements that pertain to this kind of NAWD, which include issues with healthcare benefits (#21; Healthcare System & Policy), worker retention and commitment (#37; Temporary Agency Work & Commitment), and relationship uncertainties (#45; Organizational Dependencies).

We observe that no topics on motivation, emotion, or cognition emerged with respect to service-based work. The majority of the research on this NAWD appears to be at country, societal, institutional or organizational level with little consideration of specific psychological constructs. In addition, no research has been found that targets freelancers in the digital service-based economy, such as programmers or web designers.

## Psychological health & task-based work

The final six topics in the model belong to task-based work. In this particular NAWD, topics exhibit a polarity between digital platform-mediated task-based work and traditional, analog modes of tasking. With regards to the former, topics #9 (Psychological Health of Carsharing Drivers), #39 (Online Microtasking & Wellbeing), and #42 (Sharing Economy Motivations) primarily concern carsharing (e.g. Uber), home-sharing (e.g. Airbnb), and online microtasking (e.g. Amazon Mechanical Turk), all of which are hot topics.

On the other hand, topics #52 (Day Labor & Mental Distress) and #80 (Latino Day Laborers, Alcoholism, & Discrimination) are cold topics, both of which deal with a specific mode of tasking that is associated with a particular demographic group and context (“illegal migrants” in the United States). The final topic, #64 (Online Microtasking & Mental Health (Experiments)) is a neutral topic which primarily concerns using Amazon Mechanical Turk for psychological experimentation.

Besides the motivation to participate in the sharing economy, no topics emerge that target specific psychological constructs such as emotions and cognitions. Additionally, and similarly to content-based work, we observe a lack of research on the regulatory, institutional, or sociodemographic aspects relevant to worker psychological health.

## Discussion

The general increase in research interest at the intersection of NAWDs and mental health and wellbeing is not unexpected given the digitalization-mediated explosion in the speed of transition out of traditional standard employment as well as the recent COVID-19 pandemic [106]. Nevertheless, the concentration of research in Europe & Central Asia, East Asia & the Pacific (which includes Australia), and North America may have critical consequences. Indeed, academic research outputs on work and psychology are concentrated in western, high-income countries [106, see 107,108]. However, Western, Educated, Industrialized, Rich, and Democratic (WEIRD) societies are a psychological peculiarity due to their being particularly “individualistic, self-obsessed, control-oriented, nonconformist, and analytical” [109]. Additionally, culture-gene co-evolutions dramatically influence the development of contextualized motivations, preferences, and behaviors [110,111].

This makes global generalizations of research findings from western contexts misleading and erroneous with respect to other realities [112], translating to policies, interventions, media depictions, and technological designs that do not necessarily address local needs, or worse, perpetuate existing stereotypes and injustices [106]. Furthermore, differences in institutional fragility and political stability translate to varying levels of psychological safety and resulting readiness and enthusiasm for embracing nonstandard work arrangements. In other words, those living under precarious conditions may associate NAWDs with emotional burden and ill-being rather than see opportunities for intrinsic motivation, growth, and wellbeing [3,46,113], and having such little data from dwellers of contexts such may lead to a critical underestimation of their needs and underrepresentation of their voices.

The predominance of quantitative methods may also reflect WEIRD psychology. As Lewis [106] puts it, “more recently, the research projects that have been receiving endorsements for their legitimacy […] have been projects adopting hypothetico-deductive models of science”, and are largely those produced by researchers from WEIRD backgrounds, whereas “research culture delegitimizes approaches that run counter to dominant narratives, as well as approaches that try to incorporate experiential knowledge from groups previously excluded from the historically homogeneous literature”. Other studies observe similar patterns [114,115]. In other words, research still primarily focuses on deductive approaches, rather than combinations and cycles of inductive-deductive processes. We therefore encourage researchers to pursue contextualized research outside of WEIRD settings, with research designs and methodologies that take into account the life histories, cultural evolutionary dynamics, and the life experiences of the researched [e.g. 116].

With regards to emerging research topics on mental health and wellbeing across various NAWDs, we observe that research covers general psychological health at the level of a geopolitical, cultural, or societal context, sociodemographic aspects of psychological health, psychological health in particular organizational settings and work environments, and psychological attributes, states, and traits at the personal level, such as emotions, motivations, and cognitions. This indicates that research is indeed seeing more diversification in terms of the employed levels of analysis [117]. With respect to the 11 cross-cutting topics, we find it interesting to see a growing research attention in topics of social welfare contrasted by a decrease attention to topics associated with negative conditions and psychological stigmas (e.g. depression and disability). This may be explained by the welcomed rise in academic researchers’ response to grand societal challenges [118] and the birth of the neurodiversity movement [119] and *positive psychology* as a discipline [120]. This is in alignment with Bliese et al.’s [121] observation that research on mental health in the workplace increasingly reflects larger societal trends. With topics such as racism, violent conflict, and pandemics dominating social discussions and the (western) media scape over the past years, there is no wonder that research reflects those issues. We also notice clear NAWD-specific trends, a discussion of is provided in the following four sections.

### Heroic owners take the lead

The surprising predominance of research on ownership-based work, particularly entrepreneurship, may owe to it being an established research field since the 1990s [122]. Nevertheless, a review by Stephan [74] indicated that only four articles were published at the intersection of entrepreneurship and psychological health between 1950 and 2010, yet one call for papers on *entrepreneurial wellbeing* in 2019 received over 90 submissions [123]. This explosion in research interest occurs in all four dimensions; the geopolitical, cultural, and societal context, sociodemographic status, the organizational environment, and individual psychological attributes, states, and traits.

Interestingly, the latter cluster shows a clear bias towards researching positive aspects of psychological health rather than “mental illness” or “psychological disorders”. It appears that entrepreneurship scholars have started integrating positive psychology in their research since its birth as a discipline in the early 2000s [120], which aligns with Wiklund et al. [123], who indicate that research on entrepreneurial wellbeing is presently “overlooking aspects of negative emotions”. This may be due to entrepreneurship’s image as a “savior” [124], “heroic act” [125], and “an emancipatory practice” [126], particularly with respect to technology startups and (aspiring) unicorns [127]. Interviews by Cunningham & Fraser [128] reveal that “people nowadays associate the word ‘entrepreneur’ to a colossus, forgetting that an entrepreneur is also an owner of a restaurant”.

Additionally, this may be due to the prevalence of research in WEIRD contexts, in line with Stephan et al. [113], who state that “resource-rich contexts likely enable entrepreneurs’ autonomy, allowing entrepreneurs to explore and experiment, […] thereby allowing them to self-actualize through experiencing their work as meaningful. […] Conversely, the lack of resources is a key stressor for entrepreneurs, constraining their autonomy and actions, […], limiting positive and enhancing negative wellbeing”.

The interest in ADHD and Entrepreneurship appears to have been sparked by the pioneering works of renowned researchers and top-ranking entrepreneurship journal editors. In agreement with Bliese et al. [121], this is may be an example of how influential authors shape the course of research fields and influence wide-reaching research interests. Additionally, this may be due to ADHD’s particularly positive impacts on entrepreneurial outcomes and digital product development due to its association with creativity and out-of-the-box thinking [129–131]. Hence, the study of this particular condition appears in alignment with research trends towards positive psychology and empowerment.

Ownership-based work, being the highest in autonomy and control out of all NAWDs (see Fig 1), is nevertheless associated with the illbeing and mental health issues that accompany high-pressure, high-risk lifestyles (Fig 2). While mental overload and workaholism have received their share of scholarly attention with regards to NAWDs in general, we encourage researchers to investigate those issues in specific correlation with autonomy and control in the rapidly changing digital society.

This may particularly focus on technostress and digital addiction. Generally, we were surprised to encounter such limited research that explicitly addresses the impact of technology on psychological health in ownership-based work. Automation and digitalization of processes and products may carry tremendous (psychological) benefits for owners, developers, and proficient users of those technologies [132,133]. On the other hand, they may threaten the livelihoods and income security of business owners who are unwilling or unable to embrace them [134]. Also, although we did find research on the psychological health of founders of technology companies [e.g. 135,136], investigations on the explicit role of technology in shaping their mental health and wellbeing are largely lacking. Therefore, issues such as insecurity, loneliness, powerlessness, addiction, and replaceability amongst ownership-based workers warrant further research, particularly amongst entrepreneurs in challenged, low-resource settings.

### Influencing all, but not researchers

The lack of research on content-based work is surprising given the present hype associated with the social media-associated influencer economy, especially among younger workers [e.g. 137,138]. One explanation might be that digital content creation and the influencer culture are not taken seriously as viable means of income generation by organization and management researchers [139], which demotes the phenomenon to being academically uninteresting. Therefore, research on mental health in association with digital content creators may have been limited by our choice of work-related search terms since it may not be perceived as “work” by scholars.

Indeed, only two topics pertain to digital content-based work, namely #28 (Digital Dependency & Addictive Behavior) and #63 (Digital Attention Economy & Self-Image), which are both hot topics but small in size. Given the high level of autonomy and control that this NAWD implies, albeit less than ownership-based work as payment is limited to content-production only, while also being potentially less of a stressful ordeal than business ownership as it involves less responsibility and risk-taking, we expected to encounter more research on work-life balance, self-fulfillment, and inspiration (Figs 1 and 2).

We encourage future research to address these gaps. Particularly, the role of regulatory, institutional, or sociodemographic factors on the psychological health of content creators would be vital to explore given the lack of external oversight on digital content creation [140] platforms and algorithms [132,141] combined with their wide global reach and consequences.

The “coldness” of topic #62 (Wellbeing in Talent Work) may be due to the generally declining research interest in NAWDs that are not digitally mediated, though it may also be that artistic research scholars do not use similar terms when describing the work of artists compared to the social sciences. In other words, they are often not described as freelancers, content creators, or gig workers by those who research them. Hence, articles on artist mental health, such as van Rens & Heritage [142] and Behroozi et al. [143], were not included in the topic modeling dataset, although they concern the mental health of professional circus performers and media artists, respectively.

Nevertheless, we expect an increase in research interest in wellbeing in creative content-based work in association with the current generative artificial intelligence (AI) revolution [144]. For instance, conversational AI (e.g. ChatGPT) and AI art (DALL-E) may induce feelings of insecurity, powerlessness, and replaceability amongst (artistic) content creators. Meanwhile, those state-of-the-art technologies are expected to fundamentally transform the “world of content” by “creating” more content creators [145], increasing competition and pressure in the existing market, while exacerbating the working conditions of those unable to adapt. On the other hand, tech-savvy, adaptable, and resilient content creators with enough resources may thrive due to the impressive efficiency and increasing autonomy provided by such tools. Future research is urged to investigate those topics in detail.

### Temporary service, insecure lives

We find it interesting that service-based work is formulated almost entirely as an insecure, stressful endeavor by researchers, in clear contrast to ownership-based work. This may be due to the schools of thought to which scholars belong. Project work associated with temporary contracts has been historically seen as a downgrade in comparison to traditional employment [19], and many studies on psychological health in service-based work are based on comparative analyses with standard employment [e.g. 146,147]. Additionally, this NAWD encompasses relatively low levels of worker autonomy and control yet may still be associated with high levels of responsibility and pressure (Fig 2). Hence, it may not provide the psychological advantages of ownership-based work (e.g. self-fulfillment and freedom), while still harboring psychological challenges such as stress and mental overload.

Nevertheless, wellbeing aspects of service-based work, such as its potential to offer work-life balance, may be further researched. We particularly encourage research on workers such as freelance cloud-based consultants, software developers, web designers, and online educators. Platform-mediated freelancing may open doors for financial security across borders, enabling those in low-resource environments to find high-paying clients elsewhere [148]. While this may improve worker livelihoods and wellbeing, it also promotes social inequalities, regulatory evasion, labor commodification, and algorithmic control [148]. Additionally, aspects of the current (generative) AI revolution and its threats of worker replacement and potentials to enhance worker productivity and work-life balance, warrant investigation [64,144]. Such research is encouraged at all levels, from psychological traits and states to institutional environments.

### Hot digital gigs and concerning stigmas

While the “hotness” of topics #9 (Psychological Health of Carsharing Drivers), #39 (Online Microtasking & Wellbeing), and #42 (Sharing Economy Motivations) is not surprising given the explosion in the digital platform economy over the last decade [20], research on other types of tasking such as last-mile delivery and household chores are absent in our dataset. We also find it interesting that specific psychological health aspects did not emerge as standalone topics. For instance, neither issues of income security, precarity, stress, or overload are predominant, nor are those of work-life balance and freedom. We encourage research on the emotions and cognitions of task- based workers, especially given the importance of such knowledge for enhancing their autonomy, empowerment, and life satisfaction (Fig 2). Additionally, an understanding of the intersection of regulations, organizational structures, and platform design with task-worker psychological health is needed.

However, limited research on the anxiety associated with online ratings appears in topic #9, which is unique to task-based work and is significantly associated with earnings [149]. We urge researchers to further investigate those issues, particularly with respect to women workers. Indeed, a large-scale analysis of online customer reviews on tasking platforms (e.g. TaskRabbit) shows that women generally receive lower evaluations [140], while the frequency of gender-based violence and discrimination incidences may be exacerbated with the proliferation of unregulated platform-mediated tasking amongst disadvantaged women [e.g. 150]. Dokuka et al. [151] recently show that “even flexible and distant working arrangements do not prevent the gender gap”, and Litman et al.’s [152] work shows the persistence of the gender pay gap in online tasking, necessitating more research attention on the issue.

As for topics #52 (Day Labor & Mental Distress) and #80 (Latino Day Laborers, Alcoholism, & Discrimination), we found their nature eye-opening. The fact that the only two topics entirely concerning task-based work that existed even prior to the platform economy boom specifically deal with Latino, immigrant day laborers was surprising and concerning. Those topics primarily concern alcoholism amongst those United States-based male workers and the social stigmas and discrimination they face. While this may reflect good-intentioned researcher goals of addressing socially relevant topics and media narratives (e.g. migrant integration and racism), it may reflect a clear bias towards associating Latino day laborers (i.e. “illegal” immigrants) with issues of alcoholism; a mental health issue that otherwise does not crystallize in any of the other topics in our model.

Indeed, mental health issues amongst day laborers are not limited to ethnic minorities [e.g. 153], and issues of substance use also concern NAWDs that are more associated with autonomy and control, such as technology entrepreneurship, as seen in stories from Silicon Valley [e.g. 154,155]. However, with task-based work often being associated with survivalism, informality, and lower socioeconomic standing [156,157], the stigma of addiction and substance use may be more pronounced [see 158], which seems to be perpetuated by academic researchers.

## Conclusions

In a rapidly digitalizing world where workers experience a myriad of abrupt and major changes throughout their lives, an understanding of emerging NAWDs and an in-depth analysis of their psychological impacts is vital. Along with the rise in digital platforms and disruptive technologies, the NAWD research field will continually grow, making the question of impacts on society and mental health increasingly relevant [see 159]. As “future work might be characterized by environmental uncertainty, interdependence, complexity, volatility, and ambiguity” (Gagné et al., 2022, p. 379), it is vital to understand its impacts to best counteract negative effects and promote social prosperity.

Our work therefore provides a unique conceptualization of the increasingly predominant work designs of the 21^st^ century, then proceeds with an analysis of a decade of academic literature at their intersection with mental health & wellbeing by means of an automated literature review using LDA topic modeling. We hence provide a starting point to guide future research as well as policy and strategic decisions to enhance the health and sustainability of humankind. We see our primary theoretical contribution in mapping the field and consequently inspiring future research, which can leverage this work and focus on targeting some of the many outlined gaps and reflecting on the uncovered biases. However, our review also shows that NAWDs are associated with a wide range of psychological health issues, making it highly relevant for practice.

Firstly, we recommend integrating elements of ownership-based work into platform- mediated service- and task-based work to enhance workers’ autonomy and freedom with regards to shaping and designing their activities, so that they are less caught in the invisible cage of algorithms [65]. This is in line with recent legislative initiatives [160], especially in the European Union, which aim to give content- and task-based workers more rights and thus more independence. Furthermore, our work can support managers, owners, and technologists in understanding the problems associated with digital, non-attachmental work, supporting them in adapting their processes and algorithms. While the broad scope of the paper is not suitable to suggest concrete steps for organizational re-structuring, we consider it an important step towards raising awareness on psychological health issues and inspiring change processes.

Understanding NAWD-specific psychological issues (Fig 1) is critical for promoting healthier work environments. Today’s workers are faced with a blend of enormous pressure and exciting tools and opportunities, necessitating constant adaptation, learning, and self-reinvention. This may not be possible without support from policy makers, platform designers, work providers, trained psychologists, and the peer community to enable making the best out of opportunities (Fig 2) and avoid falling behind in a highly complex world.

With respect to digital natives, NAWDs may not be perceived as major changes in the world of work. Indeed, they may never experience analogue and hierarchical work relationships and be oblivious to the risks associated with platform and algorithmic control. For new workforce joiners and beyond, our findings may serve as a reflection tool and a guide for work-related decisions and behaviors. A summary of implications for researchers and practitioners is seen in Table 3.

**Table 3:** A summary of implications for researchers and practitioners.

Implications for Researchers	Implications for Practitioners
Leverage the conceptualization of non-attachmental work designs for future studies on the future of work. *Examples*: *Expand the framework through adding NAWD subcategories while incorporating latest technological developments*, *for instance generative artificial intelligence*.	Leverage the conceptualization of non-attachmental work designs to understand and address predominant mental health in NAWDs relevant to your practice area. *Examples*: *Instigate discussions on well-being promoting work culture and practices relevant to NAWDs in your practice area*.
Pioneer studies in areas that are of high relevance to society and practice yet remain under-researched. *Examples*: *Conduct mental health research with respect to gender and platform labor*, *youth (influencers) and the content economy*, *and the dark side of business ownership*.	Raise awareness on the various facets of mental health and wellbeing that may not be stereotypically associated with a particular NAWD. *Examples*: *Educate entrepreneurs on issues of burnout and addiction*.
Take action to address the societal biases and stereotypes that may be magnified through the current research scape. *Examples*: *Focus on de-idealizing entrepreneurs and de-stigmatizing migrant freelancers while researching non-WEIRD populations*.	Integrate elements of ownership-based work into platform- mediated service- and task-based work to enhance workers’ autonomy and well-being. *Examples*: *Redesign algorithms and processes to worker digital overload*, *burnout*, *and unsafety*.

### Methodological limitations

While LDA topic modeling uncovers unique patterns and classifications in text data that may otherwise not be possible, it comes with a few limitations. Firstly, identification of the optimal number of topics is a complex endeavor, where reliance on statistical metrics alone (i.e. coherence and perplexities) may lead to shortcomings [86,87,89,90]. Therefore, we employed those metrics as a pre-selection step to identify a list of suitable models, then proceeded to a manual model comparison to select the one most appropriate for our purposes. Although this process is impacted by an unavoidable researcher bias, it combines positivistic and constructionist approaches and reduces blind dependence on context-void statistical data. Also, having chosen a larger model carries the advantage of increased topic diversity, differentiation, and informativeness, enabling a NAWD-specific analysis. We therefore urge researchers to combine both inductive and deductive approaches when conducting text analyses and avoid mere reliance on numerical metrics when evaluating topic models.

Additionally, despite our efforts to ensure that our search terms are as inclusive and representative as possible, we might have missed some essential terms with regards to some aspects of NAWDs or psychological health, resulting in biasing the dataset in one direction or the other. This might have been particularly relevant for content- and service-based work. In addition, we might have inadvertently reproduced existing biases pertaining to psychiatric and mental health terminology [see 161]. We therefore encourage future research on mental health and wellbeing pertaining to each NAWD particularly, while expanding the search terms to include those more commonly used outside of academia and not explicitly associated with income generation. Also, researchers could expand such analysis to include grey literature as well.

Furthermore, given that the dataset does not include literature from April 2022 and beyond, there could indeed be relevant research that has been missed in the analysis which would have resulted in additional topics, particularly in the wake of the current generative AI revolution, post-pandemic recovery, and the war in Europe. However, this was unavoidable given the time needed for literature search and text data download, formatting, cleanup, and preparation for topic modeling, followed by time-consuming model evaluation, content analysis, topic classification, pattern discovery, and interpretation processes. We hope that scholars build on our work through particularly researching mental health and non-attachmental work in the context of current technological advances and emerging societal crises and challenges. Finally, we wish for our work to pavee the way for living systematic reviews [162] on the topic as well as empirical studies on various aspects of the NAWD-Wellbeing nexus.

## Supporting information

S1 Appendix List of search items pertaining to NAWDs and psychological health. (DOCX)

S2 Appendix The topic summary table shows a detailed representation of the 80 topics in the chosen model. Topics with blue highlighting in the “Topic #” column are cold topics for which a trend of decreasing research interest has been seen (the darker the blue shade, the colder the topic), while those highlighted in red are hot topics for which research interest has been increasing (the darker the red, the hotter the topic). The “Brief Title” column corresponds to first-level codes based on the specific topic contents. The “Overarching Category” column indicates the second-order codes which correspond to work design (ownership-based, content-based, service-based, task-based, cross-cutting) and in one case to research design (methods). The “Representative Words” column includes five of the most common words that associate with each topic and best represent its meaning. The “Example Citation” column includes a top loading (i.e. highly correlated) scientific article with the respective topic that well exemplifies the topic’s contents, and its loading value with the respective topic is indicated in “Example Loading”. The “Topic Size” corresponds to the total number of scientific articles with a loading of ≥10% with regards to the topic. The “Marginal Topic Distribution” is a measure of the importance of a topic for the entire corpus based on the distinctiveness of the words that it includes [94].
(DOCX)

S1 Data (XLSX)
