# Supplementary material for: The blessing and curse of “no strings attached”: An automated literature analysis of psychological health and non-attachmental work in the digitalization era
Source: PLoS One. 2024 Feb 8;19(2):e0298040. doi: 10.1371/journal.pone.0298040 (PMC10852238; doi:10.1371/journal.pone.0298040)
Supplement: S1 Appendix — (DOCX) [file pone.0298040.s001.docx]

**The Blessing and Curse of “No Strings Attached”**

**An Automated Literature Analysis of Psychological Health and Non-Attachmental Work in the Digitalization Era**

***Lubna Rashid, Clemens Möckel, and Stephan Bohn***

***S1 Appendix***

**Search terms pertaining to NAWDs:**

Entrepreneur*; founder*; digital nomad*; passion economy; creator economy; content creat*; open-source develop*; cloud-based consultant*; aspirational labo[u]r; attention economy; influencer*; streamer*; shar* economy; access economy; peer*[to-peer] economy; community-based economy; collaborative economy; renting economy; on-call work*; on-demand economy; on-demand labo[u]r; odd job*; microtasking; micro[-]work*; click[-]work*; cloud[-]work*; content moderat*; human intelligence task; precarious work*; day labo[u]r*; freelanc*; self-employ*, contract work*, sub-contract*, contingent work*, agency work*, temporary work*; leased work*; gig work*; gig economy; platform work*; platform economy; platform capitalism; platform co[-]op*; crowd[ ]work*; crowd[-]sourc*; crowd-based capitalism; social networked labo[u]r; new work; nonstandard work; alternative work.

**Search terms pertaining to psychological health:**

Psychiatric disorder; psychiatric illness; psychiatric disability; mental* ill*; mental disorder; mental* disab*; mental* [in]stab*; abnormal psychology; suicid*; substance dependen*; abus*; alcohol; psychoactive; addict*; self-harm*; trauma*; ptsd; phobi*; obsessi*; compulsi*; ocd; sleep disorder; sleep-wake disorder; insomnia*; schizophreni*; delusion*; deliriu*; hallucinat*; mani*[c/a]; psychosis; psychot*; neuroti*; aberrant salience; cognitive decline; bipolar; dissociative; affective disorder; eating disorder; mood disorder; behavio[u]ral disorder; attention deficit disorder; adhd; autis*[m/tic]; asperger*; spectrum disorder; personality disorder; somatoform; depress*; panic; emotion* [in]stab*; emotional; stress*; distress; overload*; insecur*; dissatisf*; anxi*[ous/ety]; fatigue; worry; unease; guilt*; irritab*; psychosocial; social suffering; lonel*; negative affect; mental health; positive psychology; well[-]being; satisf*; quality of life; happiness; happy; thriv*[ing]; flourish*; meaning*[full/fullness]; eudaimonia; eudemonic; hedoni*[c/a]; resilien*; motivat*; self[-]esteem; positive affect
