# Supplementary material for: The blessing and curse of “no strings attached”: An automated literature analysis of psychological health and non-attachmental work in the digitalization era
Source: PLoS One. 2024 Feb 8;19(2):e0298040. doi: 10.1371/journal.pone.0298040 (PMC10852238; doi:10.1371/journal.pone.0298040)
Supplement: S2 Appendix — Topics with blue highlighting in the “Topic #” column are cold topics for which a trend of decreasing research interest has been seen (the darker the blue shade, the colder the topic), while those highlighted in red are hot topics for which research interest has been increasing (the darker the red, the hotter the topic). The “Brief Title” column corresponds to first-level codes based on the specific topic contents. The “Overarching Category” column indicates the second-order codes which correspond to work design (ownership-based, content-based, service-based, task-based, cross-cutting) and in one case to research design (methods). The “Representative Words” column includes five of the most common words that associate with each topic and best represent its meaning. The “Example Citation” column includes a top loading (i.e. highly correlated) scientific article with the respective topic that well exemplifies the topic’s contents, and its loading value with the respective topic is indicated in “Example Loading”. The “Topic Size” corresponds to the total number of scientific articles with a loading of ≥10% with regards to the topic. The “Marginal Topic Distribution” is a measure of the importance of a topic for the entire corpus based on the distinctiveness of the words that it includes [94]. (DOCX) [file pone.0298040.s002.docx]

**The Blessing and Curse of “No Strings Attached”**

**An Automated Literature Analysis of Psychological Health and Non-Attachmental Work in the Digitalization Era**

***Lubna Rashid, Clemens Möckel, and Stephan Bohn***

***S2 Appendix***

| **Topic #** | **Brief**  **Title** | **Overarching Category** | **Representative**  **Words** | **Example**  **Citation** |  | **Example Loading** | **Topic Size** | **Marginal Topic Distribution** | |
| --- | --- | --- | --- | --- | --- | --- | --- | --- | --- |
| 1 | Qualitative Methods | Methods | Interview; Experience; Qualitative; Process; Theme | von Zumbusch, J. S. H., & Lalicic, L. (2020). The role of co-living spaces in digital nomads’ well-being. In Information Technology & Tourism (Vol. 22, Issues 3, SI, pp. 439–453). https://doi.org/10.1007/s40558-020-00182-2. |  | 0.34 | 154 | 0.034 |  |
| 2 | Student Psychology | Ownership-Based | College; Education: Knowledge; Psychology; Cognition | Yu, X., & Zhang, B. (2021). Innovation Strategy of Cultivating Innovative Enterprise Talents for Young Entrepreneurs Under Higher Education. In Frontiers in Psychology (Vol. 12). https://doi.org/10.3389/fpsyg.2021.693576. |  | 0.69 | 30 | 0.011 |  |
| 3 | Identity & Meaning Generation | Ownership-Based | Identity; Disappointment; Image; Effectual; Logic | Barberá-Tomás, D., Castelló, I., de Bakker, F. G. A., & Zietsma, C. (2019). Energizing through Visuals: How Social Entrepreneurs Use Emotion-Symbolic Work for Social Change. Academy of Management Journal, 62(6), 1789–1817. https://doi.org/10.5465/amj.2017.1488. |  | 0.50 | 9 | 0.006 |  |
| 4 | Work & Family | Cross-Cutting | Family; Child; Parent; Relationship; Balance | Hanson, S. K., Hessel, H. M., & Danes, S. M. (2019). Relational processes in family entrepreneurial culture and resilience across generations. In Journal of Family Business Strategy (Vol. 10, Issue 3). https://doi.org/10.1016/j.jfbs.2018.11.001. |  | 0.53 | 20 | 0.008 |  |
| 5 | Psychological Health of Entrepreneurs | Ownership-Based | Entrepreneurs; Opportunity; Behavior; Ability; Experience | Su, X., Liu, S., Zhang, S., & Liu, L. (2020). To Be Happy: A Case Study of Entrepreneurial Motivation and Entrepreneurial Process from the Perspective of Positive Psychology. In Sustainability (Vol. 12, Issue 2). https://doi.org/10.3390/su12020584. |  | 0.28 | 92 | 0.030 |  |
| 6 | Gender & Racial Biases | Ownership-Based | Gender; Antisocial; Stereotype; Black; Persistence | Randolph, A. F., Greenberg, D., Simon, J. K., & Gartner, W. B. (2022). Exploring differences in the antisocial behaviors of adolescent rule-breaking that affect entrepreneurial persistence. In International Journal of Entrepreneurial Behavior & Research (Vol. 28, Issue 2, pp. 471–499). https://doi.org/10.1108/IJEBR-03-2021-0179. |  | 0.58 | 14 | 0.007 |  |
| 7 | Affect | Ownership-Based | Positive; Negative; Affect; Cognitive; Mood | Baron, R., Hmieleski, K., & Henry, R. (2012). Entrepreneurs’ dispositional positive affect: The potential benefits—And potential costs—Of being “up.” Journal of Business Venturing, 27(3), 310–324. https://doi.org/10.1016/j.jbusvent.2011.04.002. |  | 0.49 | 29 | 0.013 |  |
| 8 | Failure & Coping | Ownership-Based | Failure; Coping; Loss; Avoidance; Past | Corner, P. D., Singh, S., & Pavlovich, K. (2017). Entrepreneurial resilience and venture failure. International Small Business Journal: Researching Entrepreneurship, 35(6), 687–708. https://doi.org/10.1177/0266242616685604. |  | 0.39 | 19 | 0.009 |  |
| 9 | Psychological Health of Carsharing Drivers | Task-Based | Uber; Platform; Gig; Flexibility; Rating | Berger, T., Frey, C. B., Levin, G., & Danda, S. R. (2019). Uber happy? Work and well-being in the “Gig Economy”. Economic Policy, 34(99), 429–477. https://doi.org/10.1093/epolic/eiz007. |  | 0.53 | 18 | 0.008 |  |
| 10 | Challenges of Unpredictable Schedules | Service-Based | Hour; Wage; Arrangement; Independent; Nonstandard | Suleiman, A. O., Decker, R. E., Garza, J. L., Laguerre, R. A., Dugan, A. G., & Cavallari, J. M. (2021). Worker perspectives on the impact of non-standard workdays on worker and family well-being: A qualitative study. BMC Public Health, 21(1), 2230–2230. https://doi.org/10.1186/s12889-021-12265-8. |  | 0.38 | 43 | 0.014 |  |
| 11 | Life Satisfaction | Ownership-Based | Satisfaction; Leisure; Experience; Relationship; Time | Rojas, M., Watkins-Fassler, K., & Rodríguez-Ariza, L. (2022). The life satisfaction of owner-manager entrepreneurs when the business of business is not only business. Applied Research in Quality of Life. https://doi.org/10.1007/s11482-022-10035-1. |  | 0.41 | 29 | 0.012 |  |
| 12 | Emotions & Firm Growth | Ownership-Based | Performance; Growth; Succession; Crisis; Survival | Yoon, J., May, K., Kang, J. H., & Solomon, G. T. (2019). The impact of emotional self-management on benefit offerings and employment growth: An analysis of the fastest growing businesses in the United States. International Entrepreneurship and Management Journal, 15(1), 175–194. https://doi.org/10.1007/s11365-018-0542-3 |  | 0.35 | 12 | 0.008 |  |
| 13 | Self-Employment & Job Satisfaction | Cross-Cutting | Satisfaction; Autonomy; Security; Salary; Gap | Sanchez-Sanchez, N., & Namkee, A. (2018). Is job satisfaction of high-level managers and self-employed more pro-cyclical than normal employees? International Journal of Manpower, 39(6), 800–819. https://doi.org/10.1108/IJM-02-2017-0037 |  | 0.51 | 55 | 0.016 |  |
| 14 | Resilience | Ownership-Based | Resilience; Crisis; Adversity; Threat; Disaster | Martinelli, E., Tagliazucchi, G., & Marchi, G. (2018). The resilient retail entrepreneur: Dynamic capabilities for facing natural disasters. In International Journal of Entrepreneurial Behavior & Research (Vol. 24, Issues 7, SI, pp. 1222–1243). https://doi.org/10.1108/IJEBR-11-2016-0386 |  | 0.49 | 46 | 0.014 |  |
| 15 | Crowdsourcing Motivation | Cross-Cutting | Innovation; Community; Platform; Motitvation; Participation | Feng, Y., Jonathan Ye, H., Yu, Y., Yang, C., & Cui, T. (2018). Gamification artifacts and crowdsourcing participation: Examining the mediating role of intrinsic motivations. Computers in Human Behavior, 81, 124–136. https://doi.org/10.1016/j.chb.2017.12.018. |  | 0.56 | 16 | 0.008 |  |
| 16 | Female Entrepreneurship Motivation | Ownership-Based | Gender; Empowerment; Responsibility; Home; Challenge | Shastri, S., Shastri, S., & Pareek, A. (2019). Motivations and challenges of women entrepreneurs: Experiences of small businesses in Jaipur city of Rajasthan. In International Journal of Sociology and Social Policy (Vol. 39, Issues 5–6, pp. 338–355). https://doi.org/10.1108/IJSSP-09-2018-0146. |  | 0.43 | 46 | 0.013 |  |
| 17 | Entrepreneurial Stress | Ownership-Based | Stress; Demand; Workload; Well-being; Strain | Xu, F., Kellermanns, F. W., & Jin, L. (2021). Between- and within-person consequences of daily entrepreneurial stressors on discrete emotions in entrepreneurs: The moderating role of personality. In Stress and Health. https://doi.org/10.1002/smi.3118. |  | 0.33 | 48 | 0.015 |  |
| 18 | Interventions for Marginalized Groups | Ownership-Based | Intervention; Program; Substinence; Literacy; Behavioral | Tingey, L., Larzelere, F., Goklish, N., Rosenstock, S., Mayo-Wilson, L. J., Pablo, E., Goklish, W., Grass, R., Sprengeler, F., Parker, S., Ingalls, A., Craig, M., & Barlow, A. (2020). Entrepreneurial, Economic, and Social Well-Being Outcomes from an RCT of a Youth Entrepreneurship Education Intervention among Native American Adolescents. International Journal of Environmental Research and Public Health, 17(7). https://doi.org/10.3390/ijerph17072383. |  | 0.58 | 17 | 0.008 |  |
| 19 | Temporal Perspectives | Methods | Time; Task; Feeling; Efficacy; Orientation | Jiang, L., Wagner, C., & Chen, X. (2021). Taking Time into Account: Understanding Microworkers’ Continued Participation in Microtasks. Journal of the Association for Information Systems, 22(4), 893–930. https://doi.org/10.17705/1jais.00684. |  | 0.51 | 10 | 0.005 |  |
| 20 | Community Empowerment in the Global South | Cross-Cutting | Community; Local; Cultural; Cooperative; Indigenous | Otake, Y., & Hagenimana, F. (2021). Gift economy and well-being: A mode of economy playing out in recovery from Rwandan tragedies. In Sustainable Development (Vol. 29, Issue 5, pp. 930–940). https://doi.org/10.1002/sd.2185. |  | 0.53 | 23 | 0.010 |  |
| 21 | Healthcare System & Policy | Service-Based | Policy; Medical; Sickness; Rehabilitation; Insurance | Lammerts, L., Vermeulen, S. J., Schaafsma, F. G., van Mechelen, W., & Anema, J. R. (2014). Return to work of workers without a permanent employment contract, sick-listed due to a common mental disorder: Design of a randomised controlled trial. In BMC Public Health (Vol. 14). https://doi.org/10.1186/1471-2458-14-594. |  | 0.54 | 20 | 0.007 |  |
| 22 | Financial Distress | Ownership-Based | Risk; Loan; Bank; Shock; Stress | Jina, B., Cheng, K., Cary, J. C., & Huang, F. (2020). Impact of Confucianism on Compensation Structure for Founder CEOs. In International Journal of Business (Vol. 25, Issue 3, pp. 266–290). |  | 0.70 | 13 | 0.007 |  |
| 23 | Age and Disadvantage | Ownership-Based | Youth; Senior; Necessity; Transition; Skill | Tam, H., Asamoah, E., & Chan, A. (2021). Developing Social Entrepreneurship as an Intervention to Enhance Disadvantaged Young People’s Sense of Self-Worth and Career Competence in Hong Kong. Applied Research in Quality of Life, 16(6), 2497–2526. https://doi.org/10.1007/s11482-021-09917-7 |  | 0.37 | 15 | 0.006 |  |
| 24 | Entrepreneurship & Academic Institutions | Ownership-Based | Education; Learning; Competency; Training; Transfer | Kazin, P., Hagen, S., Prichislenko, A., & Zlenko, A. (2017). Developing the Entrepreneurial University through Positive Psychology and Social Enterprise: A Case Study of Curriculum Innovation in Russia. In Educational Studies Moscow (Issue 3, pp. 110–131). https://doi.org/10.17323/1814-9545-2017-3-110-131. |  | 0.40 | 46 | 0.012 |  |
| 25 | Conflict & Satisfaction in Teams | Ownership-Based | Team; Conflict; Diversity; Interdependence; Integration | Swab, R. G., Cogan, A., Pret, T., & Marshall, D. R. (2021). Examining the Creative Self-Efficacy, Goal Interdependence, and Satisfaction of New Venture Teams in the Board Game Industry. In Entrepreneurship Research Journal. https://doi.org/10.1515/erj-2021-0142. |  | 0.43 | 12 | 0.006 |  |
| 26 | Self-Employment, Precarity, & Depression | Service-Based | Depressive; Symptom; Hour; Income; Health | Kim, H., Park, J., Kim, A. R., & Kim, Y. (2021). Factors related to depressive symptoms in Korean self-employed workers. In Annals of Occupational and Environmental Medicine (Vol. 33). https://doi.org/10.35371/aoem.2021.33.e20. |  | 0.79 | 52 | 0.016 |  |
| 27 | Disability & Psychiatric Issues | Cross-Cutting | Disability; Psychiatric; Service; Support; Opportunity | Ostrow, L., Smith, C., Penney, D., & Shumway, M. (2019). “It suits my needs”: Self-employed individuals with psychiatric disabilities and small businesses. Psychiatric Rehabilitation Journal, 42(2), 121–131. https://doi.org/10.1037/prj0000341. |  | 0.55 | 23 | 0.007 |  |
| 28 | Digital Dependency & Addictive Behavior | Content-Based | Medium; Addiction; Internet; Workaholic; Enjoyment | Bhargava, V. R., & Velasquez, M. (2021). Ethics of the Attention Economy: The Problem of Social Media Addiction. Business Ethics Quarterly, 31(3), 321–359. https://doi.org/10.1017/beq.2020.32. |  | 0.58 | 11 | 0.004 |  |
| 29 | Reviews & Conceptual Models | Methods | Context; Literature; Theory; Framework; Study | Henninger, C. E., Brydges, T., Iran, S., & Vladimirova, K. (2021). Collaborative fashion consumption—A synthesis and future research agenda. In Journal of Cleaner Production (Vol. 319). https://doi.org/10.1016/j.jclepro.2021.128648 |  | 0.29 | 158 | 0.050 |  |
| 30 | Depression | Cross-Cutting | Disorder; Depression; Mental; Poor; Anxiety | Ophir, Y., Sisso, I., Asterhan, C. S. C., Tikochinski, R., & Reichart, R. (2020). The Turker blues: Hidden factors behind increased depression rates among Amazon’s Mechanical Turkers. Clinical Psychological Science, 8(1), 65–83. https://doi.org/10.1177/2167702619865973. |  | 0.37 | 40 | 0.013 |  |
| 31 | Self-Employment Panel Data | Methods | Model; Dummy; Control; Household; Regression | Rietveld, C. A., Hessels, J., & van der Zwan, P. (2015). The stature of the self-employed and its relation with earnings and satisfaction. Economics and Human Biology, 17, 59–74. https://doi.org/10.1016/j.ehb.2015.02.001 |  | 0.72 | 110 | 0.037 |  |
| 32 | Farm Labor & Household Wellbeing | Cross-Cutting | Farmer; Household; Rural; Microcredit; Security | Banna, M. H. A., Sayeed, A., Kundu, S., Kagstrom, A., Sultana, M. S., Begum, M. R., & Khan, M. S. I. (2022). Factors associated with household food insecurity and dietary diversity among day laborers amid the COVID-19 pandemic in Bangladesh. BMC Nutrition, 8(1), 25–25. https://doi.org/10.1186/s40795-022-00517-8 |  | 0.38 | 19 | 0.008 |  |
| 33 | Entrepreneurship in Crisis & Emergency | Ownership-Based | Pandemic; Disurption; Protest; Uncertainty; Violent | Viswanathan, M., Faruque Aly, H., Duncan, R., & Mandhan, N. (2021). Unequal but essential: How subsistence consumer–entrepreneurs negotiate unprecedented shock with extraordinary resilience during COVID‐19. Journal of Consumer Affairs, 55(1), 151–178. https://doi.org/10.1111/joca.12351 |  | 0.34 | 29 | 0.008 |  |
| 34 | Values & Power Dynamics | Ownership-Based | Power; Behavior; Barrier; Official; Shame | Doern, R., & Goss, D. (2014). The Role of Negative Emotions in the Social Processes of Entrepreneurship: Power Rituals and Shame-Related Appeasement Behaviors. Entrepreneurship: Theory & Practice, 38(4), 863–890. https://doi.org/10.1111/etap.12026 |  | 0.51 | 8 | 0.007 |  |
| 35 | Gender & Work-Family Conflict | Ownership-Based | Conflict; Role; Exhaustion; Work-Family; Expectation | De Clercq, D., Kaciak, E., & Thongpapanl, N. (Tek). (2021). Work-to-family conflict and firm performance of women entrepreneurs: Roles of work-related emotional exhaustion and competitive hostility. International Small Business Journal: Researching Entrepreneurship, 1. https://doi.org/10.1177/02662426211011405 |  | 0.49 | 25 | 0.011 |  |
| 36 | Work-from-Home Consequences | Cross-Cutting | Balance; Flexibility; Demand; Isolation; Home | Gold, M., & Mustafa, M. (2013). “Work always wins”: Client colonisation, time management and the anxieties of connected freelancers. New Technology, Work & Employment, 28(3), 197–211. https://doi.org/10.1111/ntwe.12017 |  | 0.50 | 16 | 0.007 |  |
| 37 | Temporary Agency Work & Committment | Service-Based | Contingent; Committment; Affective; Exhaustion; Autonomous | Chambel, M. J., Sobral, F., Espada, M., & Curral, L. (2015). Training, exhaustion, and commitment of temporary agency workers: A test of employability perceptions. European Journal of Work and Organizational Psychology, 24(1), 15–30. https://doi.org/10.1080/1359432X.2013.849246 |  | 0.65 | 26 | 0.008 |  |
| 38 | Quantitative Methods #1 | Methods | Variable; Model; Effect; Factor; Hypothesis | Al-Jubari, I. (2019). College Students’ Entrepreneurial Intention: Testing an Integrated Model of SDT and TPB. In SAGE Open (Vol. 9, Issue 2). https://doi.org/10.1177/2158244019853467 |  | 0.49 | 107 | 0.028 |  |
| 39 | Online Microtasking & Wellbeing | Task-Based | Gig; Platform; Mturk; Digital; Meaningfulness | Kost, D., Fieseler, C., & Wong, S. I. (2018). Finding meaning in a hopeless place? The construction of meaningfulness in digital microwork. In Computers in Human Behavior (Vol. 82, pp. 101–110). https://doi.org/10.1016/j.chb.2018.01.002 |  | 0.50 | 25 | 0.009 |  |
| 40 | Emotions & Self-Expression | Ownership-Based | Emotion; Expression; Joy; Display; Reappraisal | Jiang, L., Yin, D., & Liu, D. (2019). Can joy buy you money? The impact of the strength, duration, and phases of an entrepreneur’s peak displayed joy on funding performance. Academy of Management Journal, 62(6), 1848–1871. https://doi.org/10.5465/amj.2017.1423 |  | 0.61 | 19 | 0.010 |  |
| 41 | Sharing Economy Motivations | Task-Based | Host; Collaborative; Airbnb; Sustainability; Service | Thaichon, P., Surachartkumtonkun, J., Singhal, A., & Alabastro, A. (2020). Host and guest value co-creation and satisfaction in a shared economy: The case of Airbnb. Journal of Global Scholars of Marketing Science, 30(4), 407–422. https://doi.org/10.1080/21639159.2020.1752278 |  | 0.60 | 26 | 0.011 |  |
| 42 | Emotions & Cognitive Appraisals | Ownership-Based | Fear; Opportuntity; Appraisal; Exploitation; Hope | Ivanova, S., Treffers, T., & Langerak, F. (2018). Emotional paths leading to opportunity desirability and feasibility beliefs through controllability. International Small Business Journal: Researching Entrepreneurship, 36(5), 546–573. https://doi.org/10.1177/0266242617751596 |  | 0.54 | 20 | 0.009 |  |
| 43 | Proactivity, Leadership & Wellbeing | Ownership-Based | Leadership; Initiative; Transformational; Effect; Cognition | Men, L. R., Chen, Z. F., & Ji, Y. G. (2021). Cultivating Relationships with Startup Employees: The Role of Entrepreneurs’ Leadership Communication. In Management Communication Quarterly (Vol. 35, Issue 4, pp. 518–545). https://doi.org/10.1177/08933189211017918 |  | 0.51 | 11 | 0.005 |  |
| 44 | Personality Traits | Ownership-Based | Personality; Trait; Extraversion; Conscientiousness; Grit | Mieg, H. A., Bedenk, S. J., Braun, A., & Neyer, F. J. (2012). How Emotional Stability and Openness to Experience Support Invention: A Study with German Independent Inventors. Creativity Research Journal, 24(2/3), 200–207. https://doi.org/10.1080/10400419.2012.677341 |  | 0.39 | 26 | 0.008 |  |
| 45 | Organisational Dependencies | Service-Based | Relationship; Agent; Income; Uncertainty; Borrower | Terry, E., Marks, A., Dakessian, A., & Christopoulos, D. (2021). Emotional Labour and the Autonomy of Dependent Self-Employed Workers: The Limitations of Digital Managerial Control in the Home Credit Sector. In Work, Employment and Society (Vol. 36, Issue 4). https://doi.org/10.1177/0950017020979504 |  | 0.50 | 9 | 0.005 |  |
| 46 | Entrepreneurial Motivation | Ownership-Based | Entrepreneurs; Personal; Perception; Motivation; Experience | Hanafiah, M. H., Usman Yousaf, Sh., & Senik, Z. C. (2016). Satisfaction of Goals Attainment and Intention to Restructure among Malaysian SME Entrepreneurs. Journal of Entrepreneurship & Business, 4(2), 13–29. http://dx.doi.org/10.17687/JEB.0402.02 |  | 0.19 | 22 | 0.021 |  |
| 47 | Emotional Intelligence | Ownership-Based | Emotion; Intelligence; Self-Regulation; Self-Awareness; Empathy | Agarwal, M., & Satsangi, A. K. (2018). The Role of Gender in Emotional Intelligence and Entrepreneurial Self-Efficacy. IUP Journal of Management Research, 17(4), 15–31. https://doi.org/10.3390/ijerph17124511 |  | 0.36 | 42 | 0.010 |  |
| 48 | National Wellbeing & Happiness | Ownership-Based | Happiness; Country; Life; Quality; Institution | Naude, W., Ernesto Amoros, J., & Cristi, O. (2014). “Surfeiting, the appetite may sicken”: Entrepreneurship and happiness. In Small Business Economics (Vol. 42, Issues 3, SI, pp. 523–540). https://doi.org/10.1007/s11187-013-9492-x |  | 0.56 | 38 | 0.014 |  |
| 49 | Creativity & Wellbeing | Ownership-Based | Creative; Inspiration; Alertness; Artistic; Innovation | Chen, M.-H., Chang, Y.-Y., Wang, H.-Y., & Chen, M.-H. (2017). Understanding Creative Entrepreneurs’ Intention to Quit: The Role of Entrepreneurial Motivation, Creativity, and Opportunity. In Entrepreneurship Research Journal (Vol. 7, Issue 3). https://doi.org/10.1515/erj-2016-0001 |  | 0.44 | 15 | 0.007 |  |
| 50 | Subjective Wellbeing | Ownership-Based | Subjective; Well-being; Feeling; Happy; Income | Honjo, Y., Ikeuchi, K., & Nakamura, H. (2022). The Mediating Effect of Financial Motives in the Association between Entrepreneurial Experience and Subjective Well-Being: Evidence from Japan. In Applied Research in Quality of Life (Vol. 17, pp. 1043–1067). https://doi.org/10.1007/s11482-021-09947-1 |  | 0.42 | 38 | 0.014 |  |
| 51 | Quantitative Methods #2 | Methods | Item; Scale; Index; Measurement; Correlation | Laguna, M., Mielniczuk, E., & Razmus, W. (2019). Test of the Bifactor Model of Job-Related Affective Well-Being. In Europe’s Journal of Psychology (Vol. 15, Issue 2, pp. 342–357). https://doi.org/10.5964/ejop.v15i2.1632 |  | 0.53 | 48 | 0.016 |  |
| 52 | Day Labor & Mental Distress | Task-Based | Laborer; Latino; Immigrant; Drinking; Abuse | Negi, N., Siegel, J., Calderon, M., Thomas, E., & Valdez, A. (2020). “They Dumped Me Like Trash”: The Social and Psychological Toll of Victimization on Latino Day Laborers’ Lives. American Journal of Community Psychology, 65(3–4), 369–380. https://doi.org/10.1002/ajcp.12406 |  | 0.66 | 37 | 0.011 |  |
| 53 | Quantitative Methods #3 | Methods | Result; Difference; Category; Dimension; Group | González, L., & Rivarés, L. (2018). Analysis of the impact of referral-based recruitment on job attitudes and turnover in temporary agency workers. Employee Relations, 40(1), 89–105. https://doi.org/10.1108/ER-11-2016-0212 |  | 0.43 | 75 | 0.023 |  |
| 54 | Autonomy & Self-Fulfillment | Ownership-Based | Autonomy; Eudaimonic; Relatedness; Vitality; Functioning | Shir, N., Nikolaev, B. N., & Wincent, J. (2019). Entrepreneurship and well-being: The role of psychological autonomy, competence, and relatedness. In Journal of Business Venturing (Vol. 34, Issue 5). https://doi.org/10.1016/j.jbusvent.2018.05.002 |  | 0.40 | 26 | 0.011 |  |
| 55 | Psychological Capital | Ownership-Based | Success; Optimism; Confidence; Psycap; Resource | Baluku, M. M., Kikooma, J. F., Bantu, E., & Otto, K. (2018). Psychological capital and entrepreneurial outcomes: The moderating role of social competences of owners of micro-enterprises in East Africa. In Journal of Global Entrepreneurship Research (Vol. 8, Issue 1). https://doi.org/10.1186/s40497-018-0113-7 |  | 0.32 | 28 | 0.008 |  |
| 56 | Refugee & Migrant Inclusion | Ownership-Based | Refugee; Immigrant; Ethnic; Integration; Discrimination | Bosiakoh, T. A., & Tetteh, V. W. (2019). Nigerian immigrant women’s entrepreneurial embeddedness in Ghana, West Africa. In International Journal of Gender and Entrepreneurship (Vol. 11, Issues 1, SI, pp. 38–57). https://doi.org/10.1108/IJGE-05-2018-0043 |  | 0.39 | 14 | 0.007 |  |
| 57 | Wellbeing in the Hospitality & Food Industries | Ownership-Based | Tourism; Lifestyle; Guest; Quality; Development | Christou, P., Hadjielias, E., & Farmaki, A. (2021). Silence, sounds and the well-being of tourism entrepreneurs in noisy tourism workplaces. In Current Issues in Tourism (Vol. 24, Issue 18, pp. 2658–2670). https://doi.org/10.1080/13683500.2020.1858036 |  | 0.50 | 14 | 0.006 |  |
| 58 | The Psychology of Social Entrepreneurship | Ownership-Based | Social; Impact; Compassion; Mission; Community | Kim, A., Moon, C. W., Kim, S. K., Koh, Y. S., & Shin, J. (2020). An Empirical Investigation on the Psychological Antecedents of Social Entrepreneurship. Entrepreneurship Research Journal, 10(3), 1–18. https://doi.org/10.1515/erj-2017-0129 |  | 0.38 | 34 | 0.014 |  |
| 59 | Technology Adoption & Worker Wellbeing | Ownership-Based | Technology; Digital; Adoption; Usefulness; Capability | Rahman, S. A., Taghizadeh, S. K., Ramayah, T., & Alam, M. M. D. (2017). Technology acceptance among micro-entrepreneurs in marginalized social strata: The case of social innovation in Bangladesh. In Technological Forecasting and Social Change (Vol. 118, pp. 236–245). https://doi.org/10.1016/j.techfore.2017.01.027 |  | 0.41 | 21 | 0.009 |  |
| 60 | Engagement & Meaning | Cross-Cutting | Engagement; Value; Meaningful; Autonomy; Burnout | Aboobaker, N., Edward, M., & Zakkariya, K. A. (2021). Workplace spirituality, well-being at work and employee loyalty in a gig economy: Multi-group analysis across temporary vs permanent employment status. In Personnel Review. https://doi.org/10.1108/PR-01-2021-0002 |  | 0.36 | 31 | 0.012 |  |
| 61 | Old Age & Wellbeing | Ownership-Based | Old; Retirement; Retention; Status; Career | Caines, V. D., Crane, M. F., Noone, J., Griffin, B., Datta, S., & Earl, J. K. (2020). Older workers: Past, present and future. In Australian Journal of Management (Vol. 45, Issues 3, SI, pp. 425–448). https://doi.org/10.1177/0312896220918912 |  | 0.32 | 20 | 0.008 |  |
| 62 | Wellbeing in Talent Work | Content-Based | Music; Beauty; Freelance; Functioning; Achievement | Zhukov, K., & Rowley, J. (2022). Crafting successful music careers: Insights from the professional lives of Australian pianists. In Research Studies in Music Education. https://doi.org/10.1177/1321103X211034647 |  | 0.34 | 11 | 0.004 |  |
| 63 | Digital Attention Economy & Self-Image | Content-Based | Comment; Hashtags; Youtube; Influencers; Image | Williams, S. (2021). Watch out for the big girls: Black plus-sized content creators creating space and amplifying visibility in digital spaces. In Feminist Media Studies (Vol. 21, Issue 8, pp. 1360–1370). https://doi.org/10.1080/14680777.2021.2004195 |  | 0.48 | 21 | 0.007 |  |
| 64 | Online Microtasking & Mental Health (Experiments) | Task-Based | Experiment; Message; Treatment; Quality; Application | Fest, S., Kvaløy, O., Nieken, P., & Schöttner, A. (2021). How (not) to motivate online workers: Two controlled field experiments on leadership in the gig economy. Leadership Quarterly, 32(6). https://doi.org/10.1016/j.leaqua.2021.101514 |  | 0.67 | 10 | 0.008 |  |
| 65 | Culture & Social Wellbeing | Ownership-Based | Well-being; Cultural; National; Consistency; Government | Woodside, A. G., Bernal, P. M., & Coduras, A. (2016). The general theory of culture, entrepreneurship, innovation, and quality-of-life: Comparing nurturing versus thwarting enterprise start-ups in BRIC, Denmark, Germany, and the United States. Industrial Marketing Management, 53, 136–159. https://doi.org/10.1016/j.indmarman.2015.11.003 |  | 0.73 | 13 | 0.007 |  |
| 66 | Insecurity & Temporary Work | Service-Based | Contract; Insecurity; Fixed-Term; Unemployed; Agency | Mauno, S., De Cuyper, N., Kinnunen, U., & De Witte, H. (2012). Work characteristics in long-term temporary workers and temporary-to-permanent workers: A prospective study among Finnish health care personnel. In Economic and Industrial Democracy (Vol. 33, Issue 3, pp. 357–377). https://doi.org/10.1177/0143831X11408939 |  | 0.58 | 51 | 0.014 |  |
| 67 | Intrinsic Motivation | Ownership-Based | Motivation; Intrinic; Externsic; Reward; Enjoyment | Zhao, Y., Qin, Y., Zhao, X., & Shi, L. (2018). Relationship Between Entrepreneurial Motivation and Crowdfunding Success Based on Qualitative Analysis-Based on Kickstarer Website Data. In Wireless Personal Communications (Vol. 102, Issue 2, pp. 1723–1734). https://doi.org/10.1007/s11277-017-5230-z |  | 0.31 | 45 | 0.014 |  |
| 68 | Entrepreneurial Persistence | Ownership-Based | Nascent; Committment; Persistence; Resource; Sense | Dost, M., Shah, S. M. M., & Saleem, I. (2021). Mentor expectations and entrepreneurial venture creation: Mediating role of the sense of nothing to lose and entrepreneurial resilience. In Journal of Entrepreneurship in Emerging Economies. https://doi.org/10.1108/JEEE-04-2021-0136 |  | 0.46 | 22 | 0.013 |  |
| 69 | Precarious Work Lives | Cross-Cutting | Precarious; Insecurity; Quality; Health; Unemployment | Allan, B. A., Autin, K. L., & Wilkins-Yel, K. G. (2021). Precarious work in the 21st century: A psychological perspective. Journal of Vocational Behavior, 126. https://doi.org/10.1016/j.jvb.2020.103491 |  | 0.55 | 49 | 0.014 |  |
| 70 | Family Support | Ownership-Based | Owner; Success; Financial; Enterprise; Family | Nishantha, B., & Pathirana, K. P. J. M. (2014). Motivation, perceived success factors and problems of entrepreneurs: Evidence from a developing country in Asia. International Journal of Process Management & Benchmarking, 4(3), 292–304. http://dx.doi.org/10.1504/IJPMB.2014.063236 |  | 0.50 | 68 | 0.021 |  |
| 71 | ADHD | Ownership-Based | ADHD; Disorder; Impulsivity; Hyperactivity; Diagnosis | Antshel, K. M. (2018). Attention Deficit/Hyperactivity Disorder (ADHD) and Entrepreneurship. Academy of Management Perspectives, 32(2), 243–265. https://doi.org/10.5465/amp.2016.0144 |  | 0.53 | 24 | 0.009 |  |
| 72 | Social Networking | Ownership-Based | Network; Trust; Reciprocity; Relationship; Tie | KC, B., Morais, D. B., Smith, J. W., Peterson, M. N., & Seekamp, E. (2019). Using social network analysis to understand trust, reciprocity, and togetherness in wildlife tourism microentrepreneurship. Journal of Hospitality & Tourism Research, 43(8), 1176–1198. https://doi.org/10.1177/1096348019840794 |  | 0.40 | 15 | 0.007 |  |
| 73 | Self-Concept | Ownership-Based | Self-Efficacy; Self-Esteem; Ability; Confidence; Belief | Arora, P., Haynie, J. M., & Laurence, G. A. (2013). Counterfactual Thinking and Entrepreneurial Self-Efficacy: The Moderating Role of Self-Esteem and Dispositional Affect. Entrepreneurship: Theory & Practice, 37(2), 359–385. |  | 0.34 | 23 | 0.008 |  |
| 74 | Sleep & Exhaustion | Cross-Cutting | Sleep; Anxiety; Recovery; Mood; Insomnia | Sprajcer, M., Jay, S. M., Vincent, G. E., Zhou, X., Vakulin, A., Lack, L., & Ferguson, S. A. (2020). Are Individuals with Low Trait Anxiety Better Suited to On-Call Work? Clocks & Sleep, 2(4), 473–486. https://doi.org/10.3390/clockssleep2040035 |  | 0.67 | 12 | 0.006 |  |
| 75 | Passion vs. Obsession | Ownership-Based | Passion; Obsessive; Harmonious; Affective; Feeling | Kakarika, M., Biniari, M., Guillén, L., & Mayo, M. (2022). Where does the heart lie? A multistage process model of entrepreneurial passion and role identity management. Journal of Organizational Behavior. https://doi.org/10.1002/job.2605 |  | 0.57 | 23 | 0.009 |  |
| 76 | Quantitative Methods #4 | Methods | Study; Variable; Effect; Significant; Survey | Best, S., & Chinta, R. (2021). Work-life balance and life satisfaction among the self-employed. In Journal of Small Business and Enterprise Development (Vol. 28, Issue 7, pp. 995–1011). https://doi.org/10.1108/JSBED-06-2019-0186 |  | 0.36 | 405 | 0.073 |  |
| 77 | Risk & Success | Ownership-Based | Orientation; Performance; Competitive; Goal; Sustainability | Croonen, E. P. M., Brand, M. J., & Huizingh, E. K. R. E. (2016). To be entrepreneurial, or not to be entrepreneurial? Explaining differences in franchisee entrepreneurial behavior within a franchise system. In International Entrepreneurship and Management Journal (Vol. 12, Issue 2, pp. 531–553). https://doi.org/10.1007/s11365-014-0352-1 |  | 0.42 | 24 | 0.011 |  |
| 78 | Case & Discourse Analyses | Methods | Way; Practice; Discourse; Narrative; Story | Santisteban, S. C., & Jones, C. (2022). Ordinary entrepreneurial psychosis. Organization, 1. https://doi.org/10.1177/13505084221079007 |  | 0.67 | 44 | 0.015 |  |
| 79 | Institutions, Development, & Informal Economy | Ownership-Based | Informal; Poverty; Government; Well-being; Base-of-Pyramid | Mahadea, D., & Khumalo, S. (2020). Entry into and Exit from Informal Microenterprise Entrepreneurship in a South African Municipality: A Tale of Resilience. In Journal of Developmental Entrepreneurship (Vol. 25, Issue 3). https://doi.org/10.1142/S108494672050020X |  | 0.47 | 27 | 0.012 |  |
| 80 | Latino Day Laborers, Alcoholism, & Discrimination | Task-Based | Distress; Depression; Alcohol; Anxiety; Discrimintation | Organista, K. C., Jung, W., & Neilands, T. B. (2020). A Structural-Environmental Model of Alcohol and Substance-Related Sexual HIV Risk in Latino Migrant Day Laborers. AIDS and Behavior, 24(11), 3176–3191. https://doi.org/10.1007/s10461-020-02876-4 |  | 0.79 | 13 | 0.006 |  |
